# GEMINI: Integrative Exploration of Genetic Variation and Genome Annotations

**DOI:** 10.1371/journal.pcbi.1003153

**Published:** 2013-07-18

**Authors:** Umadevi Paila, Brad A. Chapman, Rory Kirchner, Aaron R. Quinlan

**Affiliations:** 1 Department of Public Health Sciences and Center for Public Health Genomics, University of Virginia, Charlottesville, Virginia, United States of America; 2 Bioinformatics Core, School of Public Health, Harvard University, Boston, Massachusetts, United States of America; University of Canterbury, New Zealand

## Abstract

Modern DNA sequencing technologies enable geneticists to rapidly identify genetic variation among many human genomes. However, isolating the minority of variants underlying disease remains an important, yet formidable challenge for medical genetics. We have developed GEMINI (GEnome MINIng), a flexible software package for exploring all forms of human genetic variation. Unlike existing tools, GEMINI integrates genetic variation with a diverse and adaptable set of genome annotations (e.g., dbSNP, ENCODE, UCSC, ClinVar, KEGG) into a unified database to facilitate interpretation and data exploration. Whereas other methods provide an inflexible set of variant filters or prioritization methods, GEMINI allows researchers to compose complex queries based on sample genotypes, inheritance patterns, and both pre-installed and custom genome annotations. GEMINI also provides methods for ad hoc queries and data exploration, a simple programming interface for custom analyses that leverage the underlying database, and both command line and graphical tools for common analyses. We demonstrate GEMINI's utility for exploring variation in personal genomes and family based genetic studies, and illustrate its ability to scale to studies involving thousands of human samples. GEMINI is designed for reproducibility and flexibility and our goal is to provide researchers with a standard framework for medical genomics.

This is a *PLOS Computational Biology* Software Article.

## Introduction

Unraveling the genetic components of human disease phenotypes demands not only accurate methods for discovering genetic variation, but also reliable strategies for interpreting the relevance of the identified variants. Owing to evermore accurate DNA sequencing technologies, human geneticists now have a potent tool for interrogating nearly every base pair in a human genome. Similarly, great algorithmic strides have been made [1], [2] for identifying single-nucleotide and insertion-deletion polymorphisms from the billions of sequenced DNA fragments. However, given the scale and complexity of these variation catalogs and the formats that describe them [3], it remains a substantial challenge to manage and interpret genome-scale variation in the context of a disease phenotype. While itself limited, we best understand the consequences of genetic variation affecting protein-coding genes. Yet as recent studies of loss-of-function variation have shown, ostensibly damaging variants are frequently artifacts of data, annotation, or analysis [4], [5]. As such, care must be taken in prioritizing potentially causal variants, even in this seemingly “simple” case. Interpretation is far more challenging in the case of non-coding variation, as we have only a preliminary grasp of the functional consequences of non-coding variation on gene regulation and fitness [6], [7], [8]. Integrating functional genomics annotations from ambitious projects such as ENCODE [9] will thus be crucial to assessing the impact of non-coding variation.

Given these analytical challenges, systematic efforts to identify genetic variation underlying disease phenotypes through exome and genome sequencing clearly depend upon the ability to assess variants in the context of the incredible wealth of both genomic and epigenomic annotations that have been curated since the completion of the human genome. The reality, however, is that this goal poses both technical and methodological challenges: genome annotation datasets are often quite large and are described in myriad file formats. Moreover, they come with varying documentation, they are frequently modified or updated, and they are housed in both centralized repositories [10], [11] and on individual laboratory websites. Substantial technical ability is consequently required for even the most basic exploratory analysis integrating diverse genome annotations; greater analytic sophistication requires intricate, lab-specific pipelines that are laborious to produce and next to impossible to *reproduce*. We argue that the human genomics community needs flexible, reproducible, and scalable software for mining genome variation in the context of crucial genome annotations.

We have therefore developed GEMINI (GEnome MINIng), a novel software package that integrates genetic variation in the VCF format [3] with both automatically installed and researcher-defined genome annotations into a unified database framework. By integrating all forms of genetic variation (i.e., SNPs, INDELs, and structural variants) with diverse genome annotations, GEMINI allows both biologists and programmers to devise custom prioritization schemes for both coding and non-coding variants that meet their research criteria. The GEMINI database system provides a powerful variant analysis framework that eliminates the need to develop complex, and often erroneous, analysis pipelines.

GEMINI provides distinct functionality that is not available with existing software. Tools such as VEP [12] and snpEff [13] exclusively predict the consequence of variation on gene transcripts. Others such as ANNOVAR [14] and our own BEDTools [15] enable one to filter variants in a VCF file based on overlaps with individual annotation files (e.g., a BED file of CpG islands). Integrating the many annotations necessary for genome-scale analyses in this manner inevitably requires custom scripts and is therefore laborious and error-prone. Other researchers have recognized this limitation and developed software that attempts to automate variant analysis and centralize genome annotations with genetic variants. However, most extant software packages are either focused primarily on applying disease association tests to variants identified among a study cohort (e.g., PLINK/SEQ, unpublished), provide a limited set of annotations, or are more difficult to use because annotations are not directly integrated with genetic variation [16]. Moreover, few existing tools allow researchers to explore genetic variation with Structured Query Language (SQL), a powerful and expressive system for data analysis. Other web-based tools such as Annotate-it [17] provide a convenient graphical interface for comparing variants to an integrated set of genome annotations. Whereas Annotate-it excels at organizing genome analysis experiments and data visualization, it has limited functionality for non-coding variation and supports neither INDELs nor structural variants.

In contrast, as described in the following sections, GEMINI extends and generalizes these basic data exploration concepts to allow researchers to: query variants and genome annotations in a common database framework using SQL, augment the database with custom annotations, prioritize variants based on sample genotypes and inheritance patterns, and develop intricate, yet reproducible analyses, using a standard database framework and programming interface. As such, GEMINI serves both as a standalone genome analysis toolkit and as a framework upon which to build sophisticated graphical analysis and visualization tools.

## Design and Implementation

### Design overview

**Figure 1:**
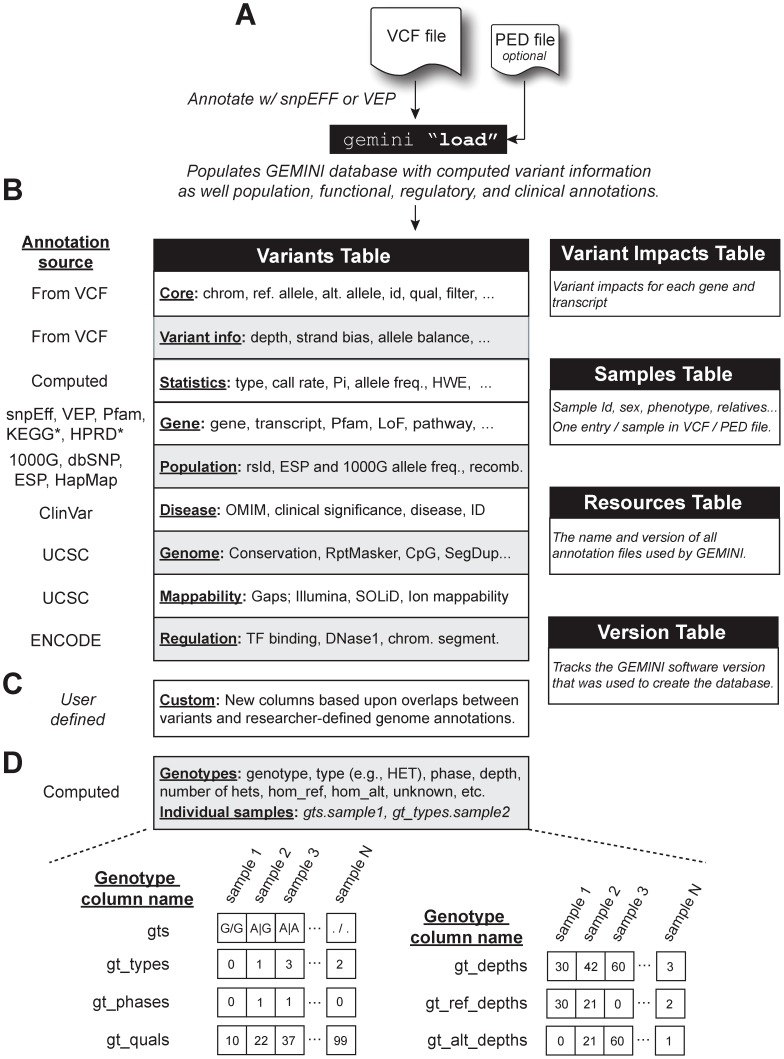
Overview of GEMINI database and annotations. (**A**) Genetic variants in the VCF format are loaded into the GEMINI database framework using the load sub-command. A PED file describing the sex, phenotype(s), and relatedness of the samples in the VCF may be provided to facilitate downstream analyses such as searches for de novo mutations or variants meeting specific inheritance patterns. (**B**) Each variant in the VCF file is annotated with information from several genome annotation sources that facilitate variant exploration and prioritization. The variants and associated annotations are stored in the variants and variant_impacts tables. (**C**) Researchers may also integrate their own annotations to facilitate custom analyses using annotations that are not pre-installed with the GEMINI software. (**D**) Genotype information for all samples is stored as compressed arrays to enable database scalability and users may access genotype information for individual samples through an enhanced SQL interface. (*) KEGG and HPRD annotations are not stored directly in the variants table, but are rather used in the context of specific GEMINI analysis tools.

As outlined in **Fig. 1**, GEMINI imports genetic variants and all sample genotypes from a VCF file into a SQLite (http://www.sqlite.org/) database (**Fig. 1A**). We prefer the use of a relational database to alternative, “NoSQL” approaches (e.g., Redis, MongoDB) because of the expressive power that SQL provides for constructing data exploration queries, its intuitive syntax, and its familiarity to many researchers. SQLite was chosen because of its speed, broad availability, and, in contrast to other database frameworks, its portability: a given GEMINI database can easily be shared as a single file among laboratory members and collaborators without a dedicated database server or additional configuration. Moreover, this portability allows researchers to “version” their research as samples and/or variant calling algorithms change by storing GEMINI databases along with the underlying VCF and sequence alignment files.

**Figure 2:**
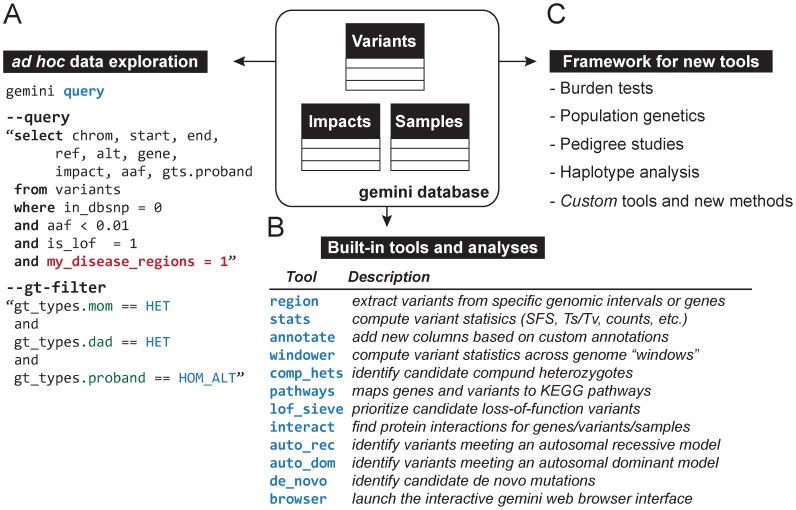
Variant mining and tool development with the GEMINI database framework. (**A**) Storing variants and annotations in the same database framework enables *ad hoc* SQL data exploration through both the query module and a Python programming interface. Analysis queries can filter variants based on pre-installed annotations (e.g., in_dbsnp = 0) and custom annotations (e.g., my_disease_regions = 1). Users may also select and filter variants based upon the genotypes of specific individuals (e.g., gt_types.mom =  = HET), thus allowing one to identify variants meeting specific inheritance patters, as shown here. (**B**) The GEMINI database framework also enables the development of tools that facilitate automated analyses for routine analysis tasks. (**C**) Moreover, it serves as a standard interface for developers to develop new tools and algorithms and to implement improved statistical tests for population and medical genetics.

Each variant in an input VCF file is extensively annotated through automatic (via Tabix [18] and pysam [19]) comparisons to a comprehensive and growing set of genomic annotation files including: dbSNP [20], ENCODE [6], ClinVar, 1000 Genomes [21], the Exome Sequencing Project [22], KEGG [23], GERP scores [24], and HPRD [25] (**Fig. 1B**; **Methods**). Annotated variants are loaded as rows in the variants database table. In the interest of reproducibility, the database also tracks (via the resources table) which version of the built-in annotations were used to create the database. Moreover, researchers may also augment the built-in annotations with custom annotation files relevant to their research (**Fig. 1C** **,** **Fig. 2A**). As we discuss in more detail below, storing extensively annotated variants in a relational database facilitates sophisticated data exploration via SQL queries and pre-defined GEMINI “tools” (a complete list of database tables and annotations are available in **Table S1**). By using a database framework, we are able to not only index variants by their genomic coordinates, but also by their associated annotations. This expedites more sophisticated queries such as, “*what are all of the novel variants that overlap CpG islands and have an alternate allele frequency greater than 5% in my cohort?*” Such functionality distinguishes GEMINI from tools such as Tabix [18] and VCFtools [3] which can either index variants solely by genomic position, or isolate specific variants by scanning the entire VCF file (which are often tens or hundreds of gigabytes in size) for desired values in the VCF format's INFO field.

### Efficient storage of sample genotype information

Studies of human disease require the ability to compare the genotypes of individual samples (e.g., cases versus controls) for each observed variant. A straightforward, yet impractical strategy for representing sample genotype information is to store the sample genotypes for each variant as distinct rows in a separate genotypes database table. In this model, accessing all observed genotypes for a given variant would thus require joining a variants table to a genotypes table, a strategy that scales very poorly when representing variation in VCF files with millions of variants and hundreds to thousands of samples. For example, merely one million variants for 1000 samples would yield 1 billion genotype rows and result in extremely poor query performance and scalability. Recognizing this limitation, we instead represent genotype information (genotype, phase, depth, etc.) for each sample as a compressed array that is stored as single column for each variant row (**Fig. 1D**). This inherently constrains the number of rows in the database to the number of variants observed. Moreover, since the proportion of rare variants will increase as a function of the number of samples, the majority of genotypes for rare variants will be identical (i.e., homozygous for the major allele) and thus highly compressible. Therefore this strategy enables both query performance and scalability while still providing necessary access to individual sample genotype information.

### Parallelization

When VCF files contain genotypes from many samples, simply reading and parsing the VCF file is time consuming. The additional cost of variant annotation causes the loading of a VCF file into GEMINI to be very computationally intensive. Therefore, in an effort to allow the loading to scale to the size of current and future VCF files including thousands of samples, the loading step can be parallelized on single machines with multiple CPUs. In addition, through use of the IPython.parallel library (http://ipython.org/ipython-doc/dev/parallel/), loading can be parallelized with computing clusters supporting LSF, Sun Grid Engine, or Torque load management systems.

### Variant annotations

Discerning the functional relationship between experimentally identified genetic variants and a phenotype demands placing variants in the context of the extensive genome annotations that have been curated since the completion of the human genome. GEMINI integrates several commonly used genome annotations directly into the SQLite database including chromosomal cytobands, CpG islands, regions under evolutionary constraint, RepeatMasker [26] annotations, segmental duplications, known assembly gaps, “mappability” scores [27], and regional recombination rates (**Fig. 1B**).

In addition, several informative variant statistics and population metrics are calculated for each variant. The rationale behind this is that the VCF format is designed to store low-level sample genotype information such as the called genotype, its likelihood, and the sequencing depth that was observed for the sample. Consequently, it is often difficult to query VCF files based on summary genotype metrics such as the count of each genotype “type” (e.g., *how many heterozygotes were observed for this variant*?), or the count of samples lacking a called genotype. In an effort to facilitate downstream variant analysis, GEMINI derives and stores these and other metrics such as deviation from Hardy-Weinberg equilibrium, inbreeding coefficients, and nucleotide diversity estimates.

### Annotating coding variation

There are now several software packages [12], [13], [14] for predicting the impact of genetic variation on protein coding transcripts. Rather than reinvent the techniques already present in these tools, GEMINI currently integrates and standardizes predictions made by either snpEff or VEP. GEMINI augments these annotations with the Pfam-A [28] protein domain that the variant affects. Each variant's clinical significance is also cataloged by comparisons to ClinVar (http://www.ncbi.nlm.nih.gov/clinvar/). Lastly, GEMINI annotates functional pathway and protein-protein interactions through built-in KEGG and HPRD catalogs, thereby permitting researchers to explore the mutational burden in pathways and interacting proteins.

### Annotating non-coding variation

Assessing the consequence of non-coding variation remains challenging, yet new insights are being made by large-scale endeavors to map human regulatory elements among hundreds of cell types [6], [8]. Nonetheless, attempting to understand non-coding variation in the context of disease requires the integration of many diverse genome annotations and exceedingly few tools exist to facilitate such research. As such, we have integrated three primary chromatin annotations from the ENCODE project: observed transcription factor binding sites [29], DNase1 hypersensitivity sites among 125 cell types [8], and Segway/ChromHMM consensus chromatin segmentation predictions among for 6 Tier 1 ENCODE cell types [9]. We anticipate continually extending and improving these annotations as dataset are made available from forthcoming efforts such as The Roadmap Epigenomics Project [30].

### Annotation file management, provenance, and reproducibility

New genome annotation files can be quickly integrated into the GEMINI framework, and since the loading step is easily parallelized, the inclusion of new or updated annotation files in the interest of empowering downstream analyses will not substantially impact the time required to load GEMINI databases. We maintain an internal record of the annotation files used by a given GEMINI version and annotation files are stored on a public server in the interest of transparency. In addition, in support of research reproducibility, we document the provenance of each annotation file as well as any post-processing that was required to modify the files for use within GEMINI: https://github.com/arq5x/gemini/tree/master/gemini/annotation_provenance.

### The GEMINI database as a framework for data exploration and tool development

Our primary motivation for directly integrating genetic variants with genome annotations is to provide a flexible framework from which to explore genetic variation for disease and population genetic studies. Integrating these data in a single database provides a standardized and consistent interface for disease genetics, data querying and exploration, and new method development. Moreover, our design allows us to adapt to evolving research needs by quickly integrating new or improved genome annotations in order to facilitate analysis and future method development.

To demonstrate the analytic utility of the database framework, we provide several built-in tools for specific analyses (**Fig. 2A,B**). The *query* tool is arguably the most powerful as it allows the researcher to compose queries against the GEMINI database that satisfy their exact research question using both pre-installed and custom annotations. For example, **Fig. 2A** demonstrates a query identifying novel, rare (<1% allele frequency), loss of function variants that meet an autosomal recessive inheritance model and overlap custom regions that are relevant to the researcher's disease of interest.

As illustrated in **Fig. 1D**, sample genotype information (e.g., the genotype, its “type” (heterozygote, homozygote, etc.), its phase, and the observed sequencing depth) is stored as database columns of compressed arrays, where each element in an array represents the relevant genotype information for a single sample. While this approach allows our database framework to easily scale to thousands of samples without generating billions of database rows, relational database systems do not inherently support queries that directly access individual genotypes. Since interrogating individual genotypes is crucial to studies of human disease, we extended the SQL syntax in GEMINI to permit queries that place conditions on individual genotypes (e.g., “SELECT gts.proband, gts.mom, gts.dad”) and filters (e.g., “gt_types.proband =  = HOM_ALT”) with a COLUMN.SAMPLE notation.

In addition, we provide other tools that address more intricate research questions without requiring the researcher to write any analysis code (**Fig. 2B**). These include tools for identifying de novo mutations, as well as variants meeting both autosomal recessive and autosomal dominant inheritance patterns in family-based studies. In order to screen for these inheritance patterns, familial relationships must be defined in an optional PED file (**Fig. 1A**), which is subsequently stored in the samples table. We further provide methods for prioritizing loss-of-function variants and identifying putative compound heterozygous variants. By integrating pathway information from KEGG and protein interaction data from HPRD, we provide tools for exploring the functional pathways that variants affect, as well as networks of interacting proteins with multiple functional variants in a given sample.

**Figure 3:**
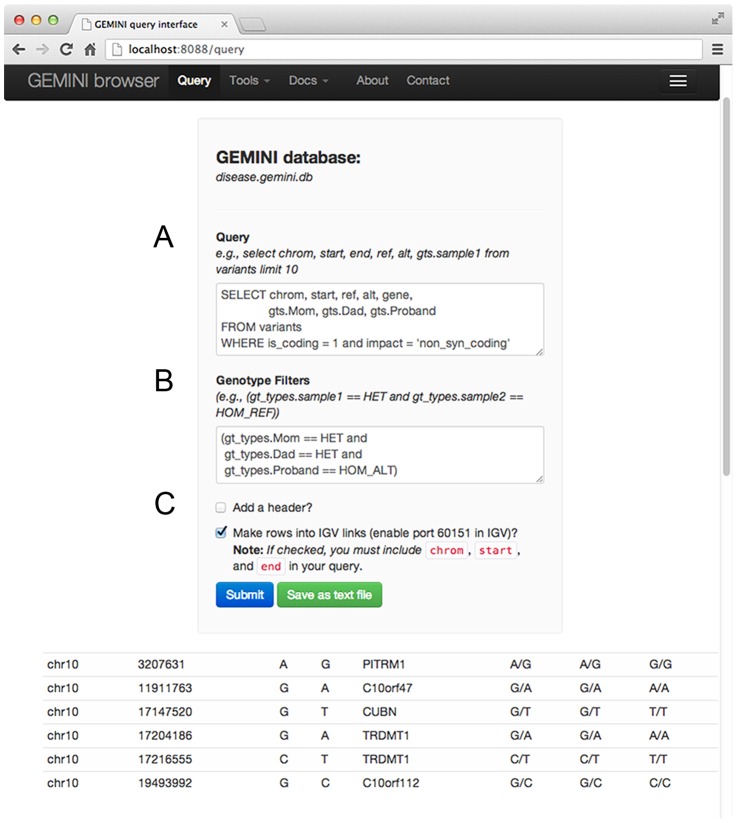
The GEMINI browser interface. In an effort to enable collaborative research and to support users who are less comfortable working on a UNIX command line, we also provide a web browser interface to GEMINI databases. This figure depicts the browser interface to the GEMINI query module; and, as illustrated in the navigation bar, interfaces also exist to other built-in analysis tools (e.g., for finding de novo mutations) and to the GEMINI documentation. (**A**) The browser interface to the query module allows users to run custom analysis queries in order to identify variants of interest. (**B**) Users may also enforce “genotype filters” that restrict the returned variants to those that meet specific genotype conditions or inheritance patterns. (**C**) Additional options are provided allowing the user to 1) add column headers describing the name of each column selected, 2) to create automatic links to the Integrative Genomics Viewer (IGV) from the reported variants, thus facilitating data exploration and validation, and 3) to report results to either the web browser or to a text file for downstream analysis.

Importantly, we enable researchers to augment the GEMINI database for their specific research needs. First, one may extend the database with genome annotations that are relevant to their own research. Secondly, researchers may create and integrate new analysis tools that leverage the GEMINI framework via Python scripts (**Methods S1**). This flexibility will allow developers to extend GEMINI as new annotations and statistical methods (e.g., gene or region based burden tests) are developed (**Fig. 2C**). Moreover, recognizing that many researchers are uncomfortable with command-line data analysis tools, we have developed an interface for accessing the above tools and their results via a web browser. The browser interface integrates documentation of the database schema and the available tools, and connects directly with the IGV genome browser [31] allowing researchers to inspect the primary DNA sequence data underlying individual variants (**Fig. 3**).

## Results

### Database loading performance

Given the size and complexity of VCF files representing variation among many samples, as well as the scale of genome annotation files, the time and resources required to import a VCF file and associated annotations into the GEMINI database were a fundamental concern in the design of the system we have developed. As such, loading a database can leverage multiple processors in order to enable reasonable database loading times. We support parallel processing on a single, multi-CPU machine and on common computing cluster frameworks (e.g., Sun Grid Engine, LSF, Torque): this inherent scalability will allow the framework to keep pace with future genetic studies involving thousands of samples. Parallel loading will enable the addition of new annotations without fearing dramatic reductions in performance. For example, using 4 processors, GEMINI required 84 minutes to load a VCF file representing 6.4 million variants detected among a CEU trio (pedigree 1463; NA12877, NA12878, NA12882) from the Illumina Platinum Genomes Project. Loading a VCF file including the genotypes of all 1092 individuals from the 1000 Genomes Project (39.7 million variants; 1092 genotypes per variant) required 28 hours using 30 processors.

### Storage requirements

Since sample genotype information is stored as compressed binary arrays in the variants table and many annotations are stored more efficiently in a SQLite database than in a text-based VCF format, the resulting GEMINI databases require substantially less storage space than the original VCF files. For example, a compressed version of the above 1000 Genomes VCF file requires 144 gigabytes after annotation with snpEff. In contrast, the corresponding GEMINI database, complete with annotations, requires just over half the space (78 gigabytes). Moreover, the compression ratio improves as the number of sample genotypes in the VCF file increases.

### Query performance

In principle, integrating genetic variation with genome annotation facilitates complex analyses, yet this goal is only satisfied through efficient database queries. Both GEMINI's built-in analysis tools and its ad hoc query interface are driven by a common query interface to the underlying SQLite database. Therefore, to assess the analytical performance of the underlying database, we have measured the time required to conduct representative ad hoc queries on GEMINI databases resulting from the trio (“GEMINI-trio”) and 1092 sample (“GEMINI-1092”) datasets above. As illustrated in **Table 1**, typical queries complete in seconds or a few minutes, regardless of whether the queries filter rows via a SQL “where” clause or via more expensive genotype filters which require decompression of the compressed sample genotype arrays. More importantly, query runtimes scale sub-linearly with respect to both the number of variants and the number of samples in the database, suggesting that our framework is well suited to typical studies of human disease. It is also important to emphasize that analytic performance can be substantially improved by conducting several queries concurrently on the same database.

**Table 1:** GEMINI query performance.

GEMINI Query	Platinum Trio Runtime (No. records)	1000 Genomes 1,092 Samples Runtime (No. records)
Return all novel variantsselect * from variants where in_dbsnp = 0	24 sec. (345,028)	11 sec. (87,939)
Return all loss-of-function variantsselect * from variants where is_lof = 1	2.1 sec. (1,126)	177 sec. (13,049)
Return all rare, loss-of-function variantsselect * from variants where is_lof = 1 and aaf <0.01	1.9 sec. (1,112)	152 sec. (12,683)
Return all loss-of-function variants and filter on a specific sample's genotype.select * from variants where is_lof = 1 --gt-filter “gt_types.NA12878 = = HET” (trio)--gt-filter “gt_types.NA20814 = = HET” (1092)	2.3 sec. (487)	194 sec. (384)

## Availability and Future Directions

We have developed a flexible new analysis framework that scales to the demands of both family-based disease studies and large-scale investigations involving thousands of individuals. By integrating genetic variation in the standard VCF format with a diverse and continually expanding set of genome annotations, GEMINI provides a uniquely powerful resource for exploring and interpreting both coding and non-coding genetic variation. Elucidating the genetic variants that underlie both unsolved single gene disorders and complex disease phenotypes requires the integration of a broad range of genome and disease annotations. Indeed a recent review of the challenges facing the interpretation of cancer genomes argues that a more detailed understanding of cancer etiology will require the integration of diverse information including pathway annotations, chromatin modifications, DNA methylation, and expression data [32]. GEMINI enables the integration of many large and heterogeneous genome annotations and as such, it provides a powerful tool to address the analytical demands of complex disease research. Therefore, we anticipate that the GEMINI framework will facilitate discovery in a broad range of research into the genetic basis of human diseases, including studies of individual genomes, unsolved Mendelian disorders, explorations of rare variants in large pedigrees, and genome-wide case-control studies. Moreover, we expect that GEMINI's portability and inherent reproducibility will allow other developers to extend the framework to create new data exploration and visualizing tools and develop novel approaches to prioritizing genetic variation in diverse contexts.

Given the clear necessity of such tools for advancing medicine in the genomic age, it is not surprising that several new commercial software packages have been developed in the last two years. Our goal is to provide a scalable, open-source medical genomics tool enabling other researchers to easily integrate new methods and genome annotations for the benefit of the human genomics research community. In future work we will continue to increase the performance of the software, expand the set of integrated genome annotations, and enhance the Python programming interface in order to facilitate tool and new method development. In addition, while we anticipate that the existing SQLite-based framework will be capable of handling tens of thousands of individuals, we will explore the alternate use of more scalable and/or distributed database systems for larger studies.

GEMINI is a freely available, open-source software package. The source code is maintained and available at: https://github.com/arq5x/gemini. Extensive documentation is available at http://gemini.readthedocs.org/ and as **Text S1**. The software is primarily implemented in Python while aspects crucial to performance are implemented in C and C++. Instructions and data files for recreating the GEMINI databases representing the Platinum Trio and the 1092 individuals from the 1000 Genomes project are available at: http://quinlanlab.cs.virginia.edu/.

## Supporting Information

Methods S1 **The GEMINI Python programming interface.**
(DOCX)

Protocol S1 **GEMINI source code, documentation, and unit test files.**
(GZ) Click here for additional data file.

Table S1 **The GEMINI database schema.**
(XLSX)

Text S1 **GEMINI user documentation.**
(PDF)
