# Supplementary material for: GEMINI: Integrative Exploration of Genetic Variation and Genome Annotations
Source: PLoS Comput Biol. 2013 Jul 18;9(7):e1003153. doi: 10.1371/journal.pcbi.1003153 (PMC3715403; doi:10.1371/journal.pcbi.1003153)
Supplement: Methods S1 — The GEMINI Python programming interface. (DOCX) [file pcbi.1003153.s001.docx]

**Supplemental Methods**

**GEMINI: integrative exploration of genetic variation and genome annotations.**

Uma Paila, Brad Chapman, Rory Kirchner, Aaron R. Quinlan*.

**The GeminiQuery Python interface.**

It is inevitable that researchers using the GEMINI framework will encounter situations where GEMINI’s available tools cannot address their exact research question. In order to address this situation, we have developed a programming interface that allows researchers to execute queries against their GEMINI database and process the results using the Python programming language. Full documentation for the interface can be found at the following URL:

<http://gemini.readthedocs.org/en/latest/content/api.html>

In the sections below, we provide example code demonstrating how the Python programming interface (specifically the GeminiQuery module) can be used to write custom, reproducible analysis scripts.

***1. One would first import the GeminiQuery module in a basic Python script.***

*#!/usr/bin/env python*

**import** sys

**from** gemini **import** GeminiQuery

***2. One would next create a GeminiQuery instance that allows one to issue queries against an existing GEMINI*** ***database.***

gemini_dbfile = sys**.**argv[1]

*# create a GeminiQery instance for the requested database*

gq **=** GeminiQuery(gemini_dbfile)

***3. Now that a connection to the database has been established, one can issue a query and iterate over the resulting rows that are returned by the query.***

*# issue a basic query and iterate over the results.*

query **=** "select chrom, start, end, gts.NA20814 from variants limit 5"

gq**.**run(query)

**for** row **in** gq:

**print** row, row['chrom'], row['gts.NA20814']

***4. One can also filter the rows that are returned by applying restrictions on sample genotype information. In the example below, we only want to process variants where sample NA20814 has a heterozygous genotype.***

*# issue a query with filters placed on genotype data.*

query **=** "select chrom, start, end, gts.NA20814 from variants limit 50"

gt_filter **=** "gt_types.NA20814 == HET"

gq**.**run(query, gt_filter)

**for** row **in** gq:

**print** row, row['chrom'], row['gts.NA20814']

***5. Access to genotype information for all samples is always available. In the example below, we use the*** sample_to_idx ***dictionary to map sample names to their respective offsets in the arrays of genotype information that are returned for each row.***

*# grab dict mapping sample to genotype array indices*

smp2idx **=** gq**.**sample_to_idx

query **=** "select chrom, start, end, gts.NA20814 from variants"

gt_filter **=** "gt_types.NA20814 == HET"

gq**.**run(query, gt_filter)

*# print a header listing the selected columns*

**print** gq**.**header

**for** row **in** gq:

*# access a NUMPY array of the sample genotypes.*

gts **=** row['gts']

*# use the sample to index dictionary to access sample genotypes*

idx **=** smp2idx['NA20814']

**print** row, gts[idx]
