# Supplementary material for: GEMINI: Integrative Exploration of Genetic Variation and Genome Annotations
Source: PLoS Comput Biol. 2013 Jul 18;9(7):e1003153. doi: 10.1371/journal.pcbi.1003153 (PMC3715403; doi:10.1371/journal.pcbi.1003153)
Supplement: Protocol S1 — GEMINI source code, documentation, and unit test files. (GZ) [file pcbi.1003153.s002.gz › gemini/gemini/views/base.j2.html]

{% block title %}{% endblock %}
{% block head %} {% endblock %}


{% block nav %}

GEMINI browser

- Query

- Tools
  - de novo mutations
  - autosomal recessive mutations
  - autosomal dominant mutations
- Docs
  - GEMINI database schema

- About
- Contact

{% endblock %}

{% block body %} {% endblock %}
