# Supplementary material for: GEMINI: Integrative Exploration of Genetic Variation and Genome Annotations
Source: PLoS Comput Biol. 2013 Jul 18;9(7):e1003153. doi: 10.1371/journal.pcbi.1003153 (PMC3715403; doi:10.1371/journal.pcbi.1003153)
Supplement: Protocol S1 — GEMINI source code, documentation, and unit test files. (GZ) [file pcbi.1003153.s002.gz › gemini/gemini/views/db_schema.j2.html]

{% extends "base.j2.html" %}
{% block title %}GEMINI query interface{% endblock %}
{% block head %}{% endblock %}
{% block body %}

- variants table
- Core VCF cols.
- Variant / PopGen cols.
- Genotype info cols
- Gene information cols.
- Optional VCF cols.
- Variant frequency cols.
- Disease info. cols.
- Genome anno. cols.
- Mappability cols.
- ENCODE info. cols.
- variant\_impacts table
- samples table

## The *variants* table

Core columns

| column\_name | type | notes |
| --- | --- | --- |
| chrom | STRING | The chromosome on which the variant resides |
| start | INTEGER | The 0-based start position. |
| end | INTEGER | The 1-based end position. |
| variant\_id | INTEGER | PRIMARY\_KEY |
| anno\_id | INTEGER | Variant transcript number for the most severely affected transcript |
| ref | STRING | Reference allele |
| alt | STRING | Alternate alele for the variant |
| qual | INTEGER | Quality score for the assertion made in ALT |
| filter | STRING | A string of filters passed/failed in variant calling |


Variant and PopGen info

|  |  |  |
| --- | --- | --- |
| type | STRING | The type of variant.  Any of: [*snp*, *indel*] |
| sub\_type | STRING | The variant sub-type.  If type is *snp*: [*ts*, (transition), *tv* (transversion)]  If type is *indel*: [*ins*, (insertion), *del* (deletion)] |
| call\_rate | FLOAT | The fraction of samples with a valid genotype |
| num\_hom\_ref | INTEGER | The total number of of homozygotes for the reference (ref) allele |
| num\_het | INTEGER | The total number of heterozygotes observed. |
| num\_hom\_alt | INTEGER | The total number of homozygotes for the reference (alt) allele |
| num\_unknown | INTEGER | The total number of of unknown genotypes |
| aaf | FLOAT | The observed allele frequency for the alternate allele |
| hwe | FLOAT | The Chi-square probability of deviation from HWE (assumes random mating) |
| inbreeding\_coeff | FLOAT | The inbreeding co-efficient that expresses the likelihood of effects due to inbreeding |
| pi | FLOAT | The computed nucleotide diversity (pi) for the site |

Genotype information

|  |  |  |
| --- | --- | --- |
| gts | BLOB | A compressed binary vector of sample genotypes (e.g., “A/A”, “A|G”, “G/G”) Access a specific sample's genotype with `gts.sample_id` |
| gt\_types | BLOB | A compressed binary vector of numeric genotype “types” (e.g., 0, 1, 2) Access a specific sample's genotype type with `gt_types.sample_id` |
| gt\_phases | BLOB | A compressed binary vector of sample genotype phases (e.g., False, True, False) Access a specific sample's genotype phasing info with `gt_phases.sample_id` |
| gt\_depths | BLOB | A compressed binary vector of the depth of aligned sequence observed for each sample Access a specific sample's sequence depth info with `gt_depths.sample_id` |


Gene information

|  |  |  |
| --- | --- | --- |
| gene | STRING | Corresponding gene name of the highly affected transcript |
| transcript | STRING | The variant transcript that was most severely affected  (for two equally affected transcripts, either the first | one is selected (VEP) or protein\_coding biotype considered (snpEff). |
| is\_exonic | BOOL | Does the variant affect an exon for >= 1transcript? |
| is\_coding | BOOL | Does the variant fall in a coding region (excl. 3’ & 5’ UTRs) for >= 1 transcript? |
| is\_lof | BOOL | Based on the value of the impact col, is the variant LOF for >= transcript? |
| exon | STRING | Exon information for the severely affected transcript |
| codon\_change | STRING | What is the codon change? |
| aa\_change | STRING | What is the amino acid change (for an snp)? |
| aa\_length | STRING | The length of CDS in terms of number of amino acids |
| biotype | STRING | The ‘type’ of the severely affected transcript (e.g.protein-coding, pseudogene, rRNA etc.) |
| impact | STRING | The consequence of the most severely affected transcript |
| impact\_severity | STRING | Severity of the highest order observed for the variant |
| polyphen\_pred | STRING | Polyphen predictions for the snps (only with VEP) for the severely affected transcript |
| polyphen\_score | FLOAT | Polyphen scores for the severely affected transcript |
| sift\_pred | STRING | SIFT predictions for the snp’s (VEP only) for the most severely affected transcript |
| sift\_score | FLOAT | SIFT scores for the predictions |
| pfam\_domain | STRING | Pfam protein domain that the variant affects |

Optional VCF INFO fields

|  |  |  |
| --- | --- | --- |
| anc\_allele | STRING | The reported ancestral allele if there is one. |
| rms\_bq | FLOAT | The RMS base quality at this position. |
| cigar | STRING | CIGAR string describing how to align an alternate allele to the reference allele. |
| depth | INTEGER | The number of aligned sequence reads that led to this variant call |
| strand\_bias | FLOAT | Strand bias at the variant position |
| rms\_map\_qual | FLOAT | RMS mapping quality, a measure of variance of quality scores |
| in\_hom\_run | INTEGER | Homopolymer runs for the variant allele |
| num\_mapq\_zero | INTEGER | Total counts of reads with mapping quality equal to zero |
| num\_alleles | INTEGER | Total number of alleles in called genotypes |
| num\_reads\_w\_dels | FLOAT | Fraction of reads with spanning deletions |
| haplotype\_score | FLOAT | Consistency of the site with two segregating haplotypes |
| qual\_depth | FLOAT | Variant confidence or quality by depth |
| allele\_count | INTEGER | Allele counts in genotypes |
| allele\_bal | FLOAT | Allele balance for hets |
| is\_somatic | BOOL | Whether the variant is somatically acquired. |

Population frequency information

|  |  |  |
| --- | --- | --- |
| in\_dbsnp | BOOL | Is this variant found in dbSnp (build 135)?  0 : Absence of the variant in dbsnp  1 : Presence of the variant in dbsnp |
| rs\_ids | STRING | A comma-separated list of rs ids for variants present in dbsnp |
| in\_hm2 | BOOL | Whether the variant was part of HapMap2. |
| in\_hm3 | BOOL | Whether the variant was part of HapMap3. |
| in\_esp | BOOL | Presence/absence of the variant in the ESP project data |
| in\_1kg | BOOL | Presence/absence of the variant in the 1000 genome project data |
| aaf\_esp\_ea | FLOAT | Minor Allele Frequency of the variant for European Americans in the ESP project |
| aaf\_esp\_aa | FLOAT | Minor Allele Frequency of the variant for African Americans in the ESP project |
| aaf\_esp\_all | FLOAT | Minor Allele Frequency of the variant w.r.t both groups in the ESP project |
| aaf\_1kg\_amr | FLOAT | Allele Frequency of the variant for samples in AMR based on AC/AN (1000g project) |
| aaf\_1kg\_asn | FLOAT | Allele frequency of the variant for samples in ASN based on AC/AN (1000g project) |
| aaf\_1kg\_afr | FLOAT | Allele frequency of the variant for samples in AFR based on AC/AN (1000g project) |
| aaf\_1kg\_eur | FLOAT | Allele Frequency of the variant for samples in EUR based on AC/AN (1000g project) |
| aaf\_1kg\_all | FLOAT | Global allele frequency (based on AC/AN) (1000g project) |

Disease phenotype info.

|  |  |  |
| --- | --- | --- |
| in\_omim | BOOL | 0 : Absence of the variant in OMIM database  1 : Presence of the variant in OMIM database |
| clinvar\_sig | STRING | The clinical significance scores for each  of the variant according to ClinVar:  *unknown*, *untested*, *non-pathogenic*  *probable-non-pathogenic*, *probable-pathogenic*  *pathogenic*, *drug-response*, *histocompatibility*  *other* |
| clinvar\_disease\_name | STRING | The name of the disease to which the variant is relevant |
| clinvar\_dbsource | STRING | Variant Clinical Channel IDs |
| clinvar\_dbsource\_id | STRING | The record id in the above database |
| clinvar\_origin | STRING | The type of variant.  Any of:  *unknown*, *germline*, *somatic*,  *inherited*, *paternal*, *maternal*,  *de-novo*, *biparental*, *uniparental*,  *not-tested*, *tested-inconclusive*,  *other* |
| clinvar\_dsdb | STRING | Variant disease database name |
| clinvar\_dsdbid | STRING | Variant disease database ID |
| clinvar\_disease\_acc | STRING | Variant Accession and Versions |
| clinvar\_in\_locus\_spec\_db | BOOL | Submitted from a locus-specific database? |
| clinvar\_on\_diag\_assay | BOOL | Variation is interrogated in a clinical diagnostic assay? |

Genome annotations

|  |  |  |
| --- | --- | --- |
| exome\_chip | BOOL | Whether an SNP is on the Illumina HumanExome Chip |
| cyto\_band | STRING | Chromosomal cytobands that a variant overlaps |
| rmsk | STRING | A comma-separated list of RepeatMasker annotations that the variant overlaps.  Each hit is of the form: name\_class\_family |
| in\_cpg\_island | BOOL | Does the variant overlap a CpG island?.  Based on UCSC: Regulation > CpG Islands > cpgIslandExt |
| in\_segdup | BOOL | Does the variant overlap a segmental duplication?.  Based on UCSC: Variation&Repeats > Segmental Dups > genomicSuperDups track |
| is\_conserved | BOOL | Does the variant overlap a conserved region?  Based on the 29-way mammalian conservation study |
| gerp\_bp\_score | FLOAT | GERP conservation score.  Only populated if the --load-gerp-bp option is used when loading.  Higher scores reflect greater conservation. **At base-pair resolution**.  Details: http://mendel.stanford.edu/SidowLab/downloads/gerp/ |
| gerp\_element\_pval | FLOAT | GERP elements P-val  Lower P-values scores reflect greater conservation. **Not at base-pair resolution**.  Details: http://mendel.stanford.edu/SidowLab/downloads/gerp/ |
| recomb\_rate | FLOAT | Returns the mean recombination rate at the variant site  Based on HapMapII\_GRCh37 genetic map |

Mappability

|  |  |  |
| --- | --- | --- |
| grc | STRING | Association with patch and fix regions from the Genome Reference Consortium:  http://www.ncbi.nlm.nih.gov/projects/genome/assembly/grc/human/  Identifies potential problem regions associated with variant calls.  Built with annotation\_provenance/make-ncbi-grc-patches.py |
| gms\_illumina | FLOAT | Genome Mappability Scores (GMS) for Illumina error models  Provides low GMS scores (< 25.0 in any technology) from:  http://sourceforge.net/apps/mediawiki/gma-bio/index.php?title=Download\_GMS  #Download\_GMS\_by\_Chromosome\_and\_Sequencing\_Technology  Input VCF for annotations prepared with:  https://github.com/chapmanb/bcbio.variation/blob/master/src/bcbio/variation/utils/gms.clj |
| gms\_solid | FLOAT | Genome Mappability Scores with SOLiD error models |
| gms\_iontorrent | FLOAT | Genome Mappability Scores with IonTorrent error models |
| in\_cse | BOOL | Is a variant in an error prone genomic position,  using CSE: Context-Specific Sequencing Errors  https://code.google.com/p/discovering-cse/  http://www.biomedcentral.com/1471-2105/14/S5/S1 |

ENCODE information

|  |  |  |
| --- | --- | --- |
| encode\_tfbs | STRING | Comma-separated list of transcription factors that were  observed by ENCODE to bind DNA in this region. Each hit in the list is constructed  as TF\_CELLCOUNT, where:  *TF* is the transcription factor name  *CELLCOUNT* is the number of cells tested that had nonzero signals.  Provenance: wgEncodeRegTfbsClusteredV2 UCSC table |
| encode\_dnaseI\_cell\_count | INTEGER | Count of cell types that were observed to have DnaseI hypersensitivity. |
| encode\_dnaseI\_cell\_list | STRING | Comma separated list of cell types that were observed to have DnaseI hypersensitivity.  Provenance: Thurman, et al, *Nature*, 489, pp. 75-82, 5 Sep. 2012 |
| encode\_consensus\_gm12878 | STRING | ENCODE consensus segmentation prediction for GM12878.    CTCF: CTCF-enriched element  E: Predicted enhancer  PF: Predicted promoter flanking region  R: Predicted repressed or low-activity region  TSS: Predicted promoter region including TSS  T: Predicted transcribed region  WE: Predicted weak enhancer or open chromatin cis-regulatory element | unknown: This region of the genome had no functional prediction. |
| encode\_consensus\_h1hesc | STRING | ENCODE consensus segmentation prediction for h1HESC. See encode\_consseg\_gm12878 for details. |
| encode\_consensus\_helas3 | STRING | ENCODE consensus segmentation prediction for Helas3. See encode\_consseg\_gm12878 for details. |
| encode\_consensus\_hepg2 | STRING | ENCODE consensus segmentation prediction for HEPG2. See encode\_consseg\_gm12878 for details. |
| encode\_consensus\_huvec | STRING | ENCODE consensus segmentation prediction for HuVEC. See encode\_consseg\_gm12878 for details. |
| encode\_consensus\_k562 | STRING | ENCODE consensus segmentation prediction for k562. See encode\_consseg\_gm12878 for details. |

## The *variant\_impacts* table

| column\_name | type | notes |
| --- | --- | --- |
| variant\_id | INTEGER | PRIMARY\_KEY (Foreign key to variants table) |
| anno\_id | INTEGER | PRIMARY\_KEY (Based on variant transcripts) |
| gene | STRING | The gene affected by the variant. |
| transcript | STRING | The transcript affected by the variant. |
| is\_exonic | BOOL | Does the variant affect an exon for this transcript? |
| is\_coding | BOOL | Does the variant fall in a coding region (excludes 3’ & 5’ UTR’s of exons)? |
| is\_lof | BOOL | Based on the value of the impact col, is the variant LOF? |
| exon | STRING | Exon information for the variants that are exonic |
| codon\_change | STRING | What is the codon change? |
| aa\_change | STRING | What is the amino acid change? |
| aa\_length | STRING | The length of CDS in terms of number of amino acids |
| biotype | STRING | The type of transcript (e.g.protein-coding, pseudogene, rRNA etc.) |
| impact | STRING | Impacts due to variation (ref.impact category) |
| impact\_severity | STRING | Severity of the impact based on the impact column value (ref.impact category) |
| polyphen\_pred | STRING | Impact of the SNP as given by PolyPhen (VEP only)  benign, possibly\_damaging, probably\_damaging, unknown |
| polyphen\_scores | FLOAT | Polyphen score reflecting severity (higher the impact, *higher* the score) |
| sift\_pred | STRING | Impact of the SNP as given by SIFT (VEP only)  neutral, deleterious |
| sift\_scores | FLOAT | SIFT prob. scores reflecting severity (Higher the impact, *lower* the score) |

## The *samples* table

| column name | type | notes |
| --- | --- | --- |
| sample\_id | INTEGER | PRIMARY\_KEY |
| name | STRING | Sample names |
| family\_id | INTEGER | Family ids for the samples [User defined, default: NULL] |
| paternal\_id | INTEGER | Paternal id for the samples [User defined, default: NULL] |
| maternal\_id | INTEGER | Maternal id for the samples [User defined, default: NULL] |
| sex | STRING | Sex of the sample [User defined, default: NULL] |
| phenotype | STRING | The associated sample phenotype [User defined, default: NULL] |
| ethnicity | STRING | The ethnic group to which the sample belongs [User defined, default: NULL] |

{% endblock %}
