# Supplementary material for: GEMINI: Integrative Exploration of Genetic Variation and Genome Annotations
Source: PLoS Comput Biol. 2013 Jul 18;9(7):e1003153. doi: 10.1371/journal.pcbi.1003153 (PMC3715403; doi:10.1371/journal.pcbi.1003153)
Supplement: Protocol S1 — GEMINI source code, documentation, and unit test files. (GZ) [file pcbi.1003153.s002.gz › gemini/gemini/views/de_novo.j2.html]

{% extends "base.j2.html" %}
{% block title %}de novo mutations{% endblock %}
{% block head %}{% endblock %}
{% block body %}

### Identify *de novo* mutations in:

*{{dbfile}}*

---

**Minimum sequence depth required (def. = 30):**

Make rows into IGV links (enable port 60151 in IGV)?

Submit

{%for row in rows %}|  |  |
| --- | --- |
|{% if igv\_links %}  |{% endif %}
{%for col in row %} {{col}} |{% endfor %}
{% endfor %}


{% endblock %}
