# Supplementary material for: GEMINI: Integrative Exploration of Genetic Variation and Genome Annotations
Source: PLoS Comput Biol. 2013 Jul 18;9(7):e1003153. doi: 10.1371/journal.pcbi.1003153 (PMC3715403; doi:10.1371/journal.pcbi.1003153)
Supplement: Protocol S1 — GEMINI source code, documentation, and unit test files. (GZ) [file pcbi.1003153.s002.gz › gemini/gemini/views/query.j2.html]

{% extends "base.j2.html" %}
{% block title %}GEMINI query interface{% endblock %}
{% block head %}{% endblock %}
{% block body %}

### GEMINI database:

*{{dbfile}}*

---

**Query**
  
*e.g., select chrom, start, end, ref, alt, gts.sample1 from variants limit 10*

{{query}}

**Genotype Filters**
  
*(e.g., (gt\_types.sample1 == HET and gt\_types.sample2 == HOM\_REF))*

{{gt\_filter}}

Add a header?

Make rows into IGV links (enable port 60151 in IGV)?
  
**Note:** *If checked, you must include* `chrom`,
`start`, and `end` in your query.

Submit

Save as text file
{%if tmp\_file%}
Click for results
{% endif %}

{% if not igv\_links\_error %}

{% for row in rows %}|  |  |
| --- | --- |
|{% if igv\_links %}  |{% endif %}
{%for col in row %} {{ row[col]}} |{% endfor %}
{% endfor %}

{% else %}

**Error:** If requesting links to IGV, you must select chrom, start, and end.

{% endif %}
{% endblock %}
