# Supplementary material for: GEMINI: Integrative Exploration of Genetic Variation and Genome Annotations
Source: PLoS Comput Biol. 2013 Jul 18;9(7):e1003153. doi: 10.1371/journal.pcbi.1003153 (PMC3715403; doi:10.1371/journal.pcbi.1003153)
Supplement: Protocol S1 — GEMINI source code, documentation, and unit test files. (GZ) [file pcbi.1003153.s002.gz › gemini/docs/templates/layout.html]

{% extends '!layout.html' %}
{%- block footer %}

{%- if show\_copyright %}
{%- if hasdoc('copyright') %}
{% trans path=pathto('copyright'), copyright=copyright|e %}© Copyright {{ copyright }}.{% endtrans %}
{%- else %}
{% trans copyright=copyright|e %}© Copyright {{ copyright }}.{% endtrans %}
{%- endif %}
{%- endif %}
{%- if last\_updated %}
{% trans last\_updated=last\_updated|e %}Last updated on {{ last\_updated }}.{% endtrans %}
{%- endif %}
{%- if show\_sphinx %}
{% trans sphinx\_version=sphinx\_version|e %}Created using Sphinx {{ sphinx\_version }}.{% endtrans %}
{%- endif %}

GPL3 licensed

{%- endblock %}
