# Supplementary material for: GEMINI: Integrative Exploration of Genetic Variation and Genome Annotations
Source: PLoS Comput Biol. 2013 Jul 18;9(7):e1003153. doi: 10.1371/journal.pcbi.1003153 (PMC3715403; doi:10.1371/journal.pcbi.1003153)
Supplement: Protocol S1 — GEMINI source code, documentation, and unit test files. (GZ) [file pcbi.1003153.s002.gz › gemini/docs/templates/page.html]

{% extends "!page.html" %}
{% set google\_analytics\_id = 'UA-39150563-1' %}
{% set disqus\_shortname = 'geminidocs' %}
{# note, currently these must be set... #}
{% set github\_base\_account = 'arq5x' %}
{% set github\_project = 'gemini' %}
{##################################################}
{# for plone-derived "edit me" & Google analytics #}
{##################################################}
{% block footer %}
{{ super() }}
{# Add plone-derived 'edit me' sticky note #}

### Edit and improve this document!

This file can be edited directly through the Web. Anyone can
update and fix errors in this document with few clicks --
no downloads needed.

1. Go to
   {{ title }}
    on GitHub.
2. **Edit** files using GitHub's text editor in your web browser (see the 'Edit' tab on the top right of the file)
3. Fill in the **Commit message** text box at the bottom of the page describing *why*
   you made the changes. Press the **Propose file change** button next to it when done.
4. Then click **Send a pull request**.
5. Your changes are now queued for review under the project's Pull requests tab on GitHub!

For an introduction to the documentation format please see the reST primer.

{% endblock %}
{#########################}
{# for disqus commenting #}
{#########################}
{% macro comments() %}Please enable JavaScript to view the comments powered by Disqus.
comments powered by Disqus
{% endmacro %}
{% block extrahead %}
{% if github\_base\_account %}
{% else %}
{% endif %}
{% if google\_analytics\_id %}
{% else %}
{% endif %}
{% endblock %}
{%- block body %}
{{ super() }}
{% if disqus\_shortname %}
{{ comments() }}
{% else %}
{% endif %}
{%- endblock %}
