# Supplementary material for: GEMINI: Integrative Exploration of Genetic Variation and Genome Annotations
Source: PLoS Comput Biol. 2013 Jul 18;9(7):e1003153. doi: 10.1371/journal.pcbi.1003153 (PMC3715403; doi:10.1371/journal.pcbi.1003153)
Supplement: Protocol S1 — GEMINI source code, documentation, and unit test files. (GZ) [file pcbi.1003153.s002.gz › gemini/docs/templates/sidebar-intro.html]

GEMINI is a flexible framework for exploring genome variation.

### GEMINI links

- Issue Tracker
- Source @ GitHub
- Mailing list @ Google Groups
- Quinlan lab @ UVa

### Sources

Browse source @ GitHub.
