# Supplementary material for: GEMINI: Integrative Exploration of Genetic Variation and Genome Annotations
Source: PLoS Comput Biol. 2013 Jul 18;9(7):e1003153. doi: 10.1371/journal.pcbi.1003153 (PMC3715403; doi:10.1371/journal.pcbi.1003153)
Supplement: Text S1 — GEMINI user documentation. (PDF) [file pcbi.1003153.s004.pdf]

gemini Documentation

Release 0.3.0b

Quinlan lab @ UVa

June 12, 2013

CONTENTS

1 Overview **1**

2 Table of contents **3**

2.1 Installation 3
2.2 Quick start 8
2.3 Annotation with snpEff or VEP 8
2.4 Loading a VCF file into GEMINI 10
2.5 Querying the GEMINI database 12
2.6 Built-in analysis tools 15
2.7 The GEMINI browser interface 26
2.8 The Gemini database schema 28
2.9 Using the GEMINI API 43
2.10 Acknowledgements 44
2.11 Release History 45
2.12 F.A.Q. 45

Python Module Index **47**

Index **49**

CHAPTER

ONE

OVERVIEW

GEMINI (GEnome MINIng) is designed to be a flexible framework for exploring genetic variation in the context of the wealth of genome annotations available for the human genome. By placing genetic variants, sample genotypes, and useful genome annotations into an integrated database framework, `GEMINI` provides a simple, flexible, yet very powerful system for exploring genetic variation for for disease and population genetics.

Using the GEMINI framework begins by loading a VCF file into a database. Each variant is automatically annotated by comparing it to several genome annotations from source such as ENCODE tracks, UCSC tracks, OMIM, dbSNP, KEGG, and HPRD. All of this information is stored in portable SQLite database that allows one to explore and interpret both coding and non-coding variation using "off-the-shelf" tools or an enhanced SQL engine.

Note:

1. GEMINI solely supports human genetic variation mapped to build 37 (aka hg19) of the human genome.
2. GEMINI is very strict about adherance to VCF format 4.1.
3. For best performance, load and query GEMINI databases on the fastest hard drive to which you have access.

CHAPTER

TWO

TABLE OF CONTENTS

2.1 Installation

2.1.1 Automated installation

GEMINI contains an automated installation script which installs GEMINI along with required Python dependencies, third party software and data files.

```
$ wget https://raw.github.com/arq5x/gemini/master/gemini/scripts/gemini_install.py
$ python gemini_install.py /usr/local /usr/local/share/gemini
```

This installs the GEMINI executable as `/usr/local/bin/gemini`, other required third party dependencies in `/usr/local/bin`, and associated data files in `/usr/local/share/gemini`. It allows easy upgrading of GEMINI and data files to the latest released version with:

```
$ gemini update
```

The installer requires Python 2.7.x, git, and the ability to ssh to your local machine. It also has options to install in "non-root" environments:

```
$ python gemini_install.py ~/gemini ~/gemini --nosudo
```

At this point, you will have a self-contained installation of GEMINI, including both the software and its associated genome annotations. However, if you have done a custom install in a "non-root" enviornment, you will first need to update your `PATH` environment variable to include the path to the bin directory that you just created by running the automated installer.

For example, if, as above, you placed you custom install in `~/gemini`, you would need to update your `PATH` as follows:

```
$ export PATH=$PATH:~/gemini/bin
```

Note that this change will only last for the life of your current terminal session. To make this more permanent, update your `.bash_profile` so that this change is made each time you login.

If successful, you should be able to run the following command from anywhere on your system:

```
$ gemini -v
gemini 0.3.0b
```

Tip: Some tips and tricks for installation issues:

1. Some older versions of wget have certificate problems with GitHub files. If you run into this problem, you can alternatively download the install script using‘‘wget –no-check-certificates‘‘ or `curl -O`.

2. The installation script is idempotent and you can re-run it multiple times without any issues. If you experience internet connectivity or other transient errors during installation, a re-run can often solve the problem (fingers crossed).

2.1.2 Software dependencies

GEMINI depends upon several widely-used genomics command line software as well as multiple Python packages. We recognize that the dependency stack is quite deep and are working on ways to minimize dependencies in the interest of the most streamlined installation process possible. Nonetheless, the following are core dependencies:

1. Python 2.7.x
2. grabix
3. samtools
4. tabix
5. bedtools
6. pybedtools

2.1.3 Manual installation

Once the above dependencies have been installed, one can begin installing `GEMINI` itself. To install you should download the latest source code from GitHub, either by going to:

```
http://github.com/arq5x/gemini
```

and clicking on "Downloads", or by cloning the git repository with:

```
$ git clone https://github.com/arq5x/gemini.git
```

Once you have the source code, run:

```
$ cd gemini
$ sudo python setup.py install
```

to install it. If you don't have permission to install it in the default directory, you can simply build the source in-place and use the package from the git repository:

```
$ python setup.py build_ext --inplace
```

2.1.4 Installing annotation files

One of the more appealing features in `GEMINI` is that it automatically annotates variants in a VCF file with several genome annotations. However, you must first install these data files on your system. It's easy enough — you just need to run the following script and tell it in which what full path you'd like to install the necessary data files. The recommended path is `/usr/local/share`, but you can install the data files wherever you want.

```
$ python gemini/install-data.py /usr/local/share/
```

2.1.5 Running the testing suite

GEMINI comes with a full test suite to make sure that everything has installed correctly on your system. We **strongly** encourage you to run these tests.

```
$ bash master-test.sh
```

Functional annotation tools

GEMINI depends upon external tools to predict the functional consequence of variants in a VCF file. We currently support annotations produced by both SnpEff and VEP. Recommended instructions for annotating existing VCF files with these tools are available here. In addition, we have attempted to standardize the terms used to describe the functional consequence of a given variant, as each annotation tool uses different vocabulary.

The variant consequence columns in the variant table are populated either by *snpEff* or *VEP* as defined by the user using the *-t* option while running pop load (To populate these columns the input VCF file should have been annotated either by *snpEff* or *VEP*):

```
$ gemini load -v my.vcf -t VEP -d my.db
$ gemini load -v my.vcf -t snpEFF -d my.db
```

By default the following columns in the variant table would be set to null:

- anno_id
- gene
- affected_gene
- affected_transcript
- affected_exon
- is_exonic
- is_lof
- is_coding
- codon_change
- aa_change
- aa_length
- biotype
- most_severe_impact
- impact_severity
- polyphen_pred
- polyphen_score
- sift_pred
- sift_score

Impacts

The table below shows the alternate *GEMINI* terms for the consequences from *snpEff* and *VEP*, for SQL queries. The last column represents the severity terms associated with the impacts:

Gemini terms	snpEff terms	VEP terms	Im
splice_acceptor	SPLICE_SITE_ACCEPTOR	splice_acceptor_variant	HI
splice_donor	SPLICE_SITE_DONOR	splice_donor_variant	HI
stop_gain	STOP_GAINED	stop_gained	HI
stop_loss	STOP_LOST	stop_lost	HI
frame_shift	FRAME_SHIFT	frameshift_variant	HI
start_loss	START_LOST	null	HI
exon_deleted	EXON_DELETED	null	HI
non_synonymous_start	NON_SYNONYMOUS_START	null	HI
non_syn_coding	NON_SYNONYMOUS_CODING	missense_variant	ME
inframe_codon_gain	CODON_INSERTION	inframe_insertion	ME
inframe_codon_loss	CODON_DELETION	inframe_deletion	ME
inframe_codon_change	CODON_CHANGE	null	ME
codon_change_del	CODON_CHANGE_PLUS_CODON_DELETION	null	ME
codon_change_ins	CODON_CHANGE_PLUS_CODON_INSERTION	null	ME
UTR_5_del	UTR_5_DELETED	null	ME
UTR_3_del	UTR_3_DELETED	null	ME
other_splice_variant	null	splice_region_variant	ME
mature_miRNA	null	mature_miRNA_variant	ME
regulatory_region	null	regulatory_region_variant	ME
TF_binding_site	null	TF_binding_site_variant	ME
regulatory_region_ablation	null	regulatory_region_ablation	ME
regulatory_region_amplification	null	regulatory_region_amplification	ME
TFBS_ablation	null	TFBS_ablation	ME
TFBS_amplification	null	TFBS_amplification	ME
synonymous_stop	SYNONYMOUS_STOP	stop_retained_variant	LO
synonymous_coding	SYNONYMOUS_CODING	synonymous_variant	LO
UTR_5_prime	UTR_5_PRIME	5_prime_UTR_variant	LO
UTR_3_prime	UTR_3_PRIME	3_prime_UTR_variant	LO
intron	INTRON	intron_variant	LO
CDS	CDS	coding_sequence_variant	LO
upstream	UPSTREAM	upstream_gene_variant	LO
downstream	DOWNSTREAM	downstream_gene_variant	LO
intergenic	INTERGENIC, INTERGENIC_CONSERVED	intergenic_variant	LO
intragenic	INTRAGENIC	null	LO
gene	GENE	null	LO
transcript	TRANSCRIPT	null	LO
exon	EXON	null	LO
start_gain	START_GAINED	null	LO
synonymous_start	SYNONYMOUS_START	null	LO
intron_conserved	INTRON_CONSERVED	null	LO
nc_transcript	null	nc_transcript_variant	LO
NMD_transcript	null	NMD_transcript_variant	LO
transcript_codon_change	null	initiator_codon_variant	LO
incomplete_terminal_codon	null	incomplete_terminal_codon_variant	LO
nc_exon	null	non_coding_exon_variant	LO
transcript_ablation	null	transcript_ablation	LO
transcript_amplification	null	transcript_amplification	LO
feature elongation	null	feature elongation	LO
feature truncation	null	feature truncation	LO

Note: "null" refers to the absence of the corresponding term in the alternate database

2.2 Quick start

gemini is designed to allow researchers to explore genetic variation contained in a VCF file. The basic workflow for working with *gemini* is outlined below.

2.2.1 Importing VCF files into gemini.

Assuming you have a valid VCF file produced by standard variation discovery programs (e.g., GATK, FreeBayes, etc.), one loads the VCF into the gemini framework with the **load** submodule:

```
$ gemini load -v my.vcf my.db
```

In this step, *gemini* reads and loads the my.vcf file into a SQLite database named my.db, whose structure is described here. While loading the database, *gemini* computes many additional population genetics statistics that support downstream analyses. It also stores the genotypes for each sample at each variant in an efficient data structure that minimizes the database size.

Loading is by far the slowest aspect of gemini. Using multiple CPUs can greatly speed up this process.

```
$ gemini load -v my.vcf --cores 8 my.db
```

2.2.2 Querying the *gemini* database.

If you are familiar with SQL, `gemini` allows you to directly query the database in search of interesting variants via the *-q* option. For example, here is a query to identify all novel, loss-of-function variants in your database:

```
$ gemini query -q "select * from variants where is_lof = 1 and in_dbsnp = 0" my.db
```

Or, we can ask for all variants that substantially deviate from Hardy-Weinberg equilibrium:

```
$ gemini query -q "select * from variants where hwe < 0.01" my.db
```

2.3 Annotation with snpEff or VEP

2.3.1 Stepwise installation and usage of VEP

Download the latest version of Variant Effect Predictor "standalone Perl script" from the Ensembl CVS server. For example:

```
$ open http://useast.ensembl.org/info/docs/variation/vep/index.html
```

Untar the tarball into the current directory.

```
$ tar -zxvf variant_effect_predictor.tar.gz
```

This will create the variant_effect_predictor directory. Now do the following:

```
$ cd variant_effect_predictor
$ perl INSTALL.pl [options]
```

By default this would install the bioperl-1.2.3, the cache files (in the .vep sub-directory of the users home directory) and the latest version of the Ensembl API (68) (in the `variant_effect_predictor` directory under a sub-directory

Bio). This script is useful for those who do not have all the modules in their system required by VEP, specifically *DBI* and *DBI::mysql*. Use this link for alternate options of the installer script.

Users (e.g mac users) who have a problem installing through this script should go for a manual installation of the latest Ensembl API (68) and bioperl-1.2.3 and follow all other installation instructions here.

The appropriate pre-build caches should be downloaded to the *.vep* directory under home from this link.

To use the cache, the gzip and `zcat` utilities are required. VEP uses `zcat` to decompress cached files. For systems where zcat may not be installed or may not work, the following option needs to be added along with the `--cache` option:

```
--compress "gunzip -c"
```

You may run the script as:

```
$ perl variant_effect_predictor.pl [OPTIONS]
```

We recommend running VEP with the following options as currently we support VEP fields specified as below:

```
$ perl variant_effect_predictor.pl -i example.vcf \
   --cache --compress "gunzip -c" \
   --terms so \
   --sift b \
   --polyphen b \
   --hgnc \
   --numbers \
   -o output \
   --vcf \
   --fields Consequence,Codons,Amino_acids,Gene,HGNC,Feature,EXON,PolyPhen,SIFT
```

A documentation of the specified options for VEP may be found at http://www.ensembl.org/info/docs/variation/vep/vep_script.html

2.3.2 Stepwise installation and usage of SnpEff

Note: Basic Requirements: Java v1.6 or later; at least 2GB of memory

Go to home directory and download the SnpEff version >=3.0. For example:

```
$ wget http://sourceforge.net/projects/snpeff/files/snpEff_v3_0_core.zip
```

Note: SnpEff should be installed preferably in `snpEff` directory in your home directory. Else, you must update the `data_dir` parameter in your snpEff.config file. For e.g. if the installation of snpEff has been done in `~/src` instead of `~/` then change the data_dir parameter in snpEff.config to `data_dir = ~/src/snpEff/data/`

Unzip the downloaded package.

```
$ unzip snpEff_v3_0_core.zip
```

Change to the `snpEff` directory and download the genome database.

```
$ cd snpEff_v3_0_core
$ java -jar snpEff.jar download GRCh37.66
```

Unzip the downloaded genome database. This will create and place the genome in the 'data' directory

```
$ unzip snpEff_v3_2_GRCh37.66.zip
```

To annotate a vcf using snpEff, use the following command:

Note: Memory options for the run may be specified by `-Xmx2G` (2GB) or Xmx4G (4GB) based on the requirement

```
$ java -Xmx4G -jar snpEff.jar -i vcf -o vcf GRCh37.66 example.vcf > example_snpeff.vcf
```

If running from a directory different from the installation directory, the complete path needs to be specified as, e.g.:

```
$ java -Xmx4G -jar path/to/snpEff/snpEff.jar -c path/to/snpEff/snpEff.config GRCh37.66 path/to/exampl
```

2.4 Loading a VCF file into GEMINI

2.4.1 Annotate with snpEff or VEP

Note: Annotate your VCF with SnpEff/VEP, prior to loading it into GEMINI, otherwise the gene/transcript features would be set to None.

GEMINI supports gene/transcript level annotations (we do not use pre-computed values here) from snpEff and VEP and hence we suggest that you first annotate your VCF with either of these tools, prior to loading it into GEMINI. The related database columns would be populated, which would otherwise be set to None if an unannotated VCF file is loaded into GEMINI.

Note: Choose the annotator as per your requirement! Some gene/transcript annotations are available with only one tool (e.g. `Polyphen/Sift` with VEP and `amino_acid length/biotype` with SnpEff). As such these values would be set to None, if an alternate annotator is used during the load step.

Instructions for installing and running these tools can be found in the following section:

Annotation with snpEff or VEP

2.4.2 The basics

Before we can use GEMINI to explore genetic variation, we must first `load` our VCF file into the GEMINI database framework. We expect you to have first annotated the functional consequence of each variant in your VCF using either VEP or snpEff (Note that v3.0+ of snpEff is required to track the amino acid length of each impacted transcript). Logically,the loading step is done with the `gemini load` command. Below are two examples based on a VCF file that we creatively name my.vcf. The first example assumes that the VCF has been pre-annotated with VEP and the second assumes snpEff.

```
# VEP-annotated VCF
$ gemini load -v my.vcf -t VEP my.db

# snpEff-annotated VCF
$ gemini load -v my.vcf -t snpEff my.db
```

As each variant is loaded into the `GEMINI` database framework, it is being compared against several annotation files that come installed with the software. We have developed an annotation framework that leverages tabix, bedtools, and pybedtools to make things easy and fairly performant. The idea is that, by augmenting VCF files with many

informative annotations, and converting the information into a `sqlite` database framework, `GEMINI` provides a flexible database-driven API for data exploration, visualization, population genomics and medical genomics. We feel that this ability to integrate variation with the growing wealth of genome annotations is the most compelling aspect of `GEMINI`. Combining this with the ability to explore data with SQL using a database design that can scale to 1000s of individuals (genotypes too!) makes for a nice, standardized data exploration system.

2.4.3 Using multiple CPUS for loading

Now, the loading step is very computationally intensive and thus can be very slow with just a single core. However, if you have more CPUs in your arsenal, you specify more cores. This provides a roughly linear increase in speed as a function of the number of cores. On our local machine, we are able to load a VCF file derived from the exomes of 60 samples in about 10 minutes. With a single core, it takes a few hours.

Note: Using multiple cores requires that you have both the `bgzip` tool from tabix and the grabix tool installed in your PATH.

```
$ gemini load -v my.vcf -t snpEff --cores 20 my.db
```

2.4.4 Using LSF, SGE and Torque clusters

Thanks to some great work from Brad Chapman and Rory Kirchner, one can also load VCF files into GEMINI in parallel using many cores on LSF, SGE or Torque clusters. One must simply specify the type of job scheduler your cluster uses and the queue name to which your jobs should be submitted.

For example, let's assume you use LSF and a queue named `preempt_everyone`. Here is all you need to do:

```
$ gemini load -v my.vcf \
         -t snpEff \
         --cores 50 \
         --lsf-queue preempt_everyone \
         my.db
```

If you use SGE, it would look like:

```
$ gemini load -v my.vcf \
         -t snpEff \
         --cores 50 \
         --sge-queue preempt_everyone \
         my.db
```

If you use Torque, it would look like: (you guessed it):

```
$ gemini load -v my.vcf \
         -t snpEff \
         --cores 50 \
         --torque-queue preempt_everyone \
         my.db
```

2.4.5 Describing samples with a PED file

GEMINI also accepts PED files in order to establish the familial relationships and phenotypic information of the samples in the VCF file.

```
$ gemini load -v my.vcf -p my.ped -t snpEff my.db
```

2.4.6 Load GERP base pair conservation scores

By default, GERP scores at base pair resolution are not computed owing to the roughly 2X increasing in loading time. However, one can optionally ask GEMINI to compute these scores by using the `--load-gerp-bp` option.

```
$ gemini load -v my.vcf --load-gerp-bp -t snpEff my.db
```

2.4.7 Loading VCFs without genotypes.

To do.

2.5 Querying the GEMINI database

The real power in the `GEMINI` framework lies in the fact that all of your genetic variants have been stored in a convenient database in the context of a wealth of genome annotations that facilitate variant interpretation. The expressive power of SQL allows one to pose intricate questions of one's variation data.

Note: If you are unfamiliar with SQL, sqlzoo has a decent online tutorial describing the basics. Really all you need to learn is the SELECT statement, and the examples below will give you a flavor of how to compose base SQL queries against the GEMINI framework.

2.5.1 Basic queries

GEMINI has a specific tool for querying a gemini database that has been `load``ed using the ``gemini load` command. That's right, the tool is called `gemini query`. Below are a few basic queries that give you a sense of how to interact with the gemini database using the `query` tool.

1. Extract all transitions with a call rate > 95%

```
$ gemini query -q "select * from variants \
                      where sub_type = 'ts' \
                      and call_rate >= 0.95" my.db
```

2. Extract all loss-of-function variants with an alternate allele frequency < 1%:

```
$ gemini query -q "select * from variants \
                      where is_lof = 1 \
                      and aaf >= 0.01" my.db
```

3. Extract the nucleotide diversity for each variant:

```
$ gemini query -q "select chrom, start, end, pi from variants" my.db
```

4. Combine `GEMINI` with `bedtools` to compute nucleotide diversity estimates across 100kb windows:

```
$ gemini query -q "select chrom, start, end, pi from variants \
                      order by chrom, start, end" my.db | \
  bedtools map -a hg19.windows.bed -b - -c 4 -o mean
```

2.5.2 Selecting sample genotypes

The above examples illustrate *ad hoc* queries that do not request or filter upon the genotypes of individual samples. Since GEMINI stores the genotype information for each variant in compressed arrays that are stored as BLOBs in the database, standard SQL queries cannot directly access individual genotypes. However, we have enhanced the SQL syntax to support such queries with C "struct-like" access. For example, to retrieve the alleles for a given sample's (in this case, sample 1094PC0009), one would add `gts.1094PC0009` to the select statement.

Here is an example of selecting the genotype alleles for four different samples (note the examples below use the test.snpEff.vcf.db file that is created in the ./test directory when you run the *bash master-test.sh* command as described above):

```
$ gemini query -q "select chrom, start, end, ref, alt, gene, \
                              gts.1094PC0005, \
                              gts.1094PC0009, \
                              gts.1094PC0012, \
                              gts.1094PC0013 \
                       from variants" test.snpEff.vcf.db

chr1        30547   30548   T       G       FAM138A ./.     ./.     ./.     ./.
chr1        30859   30860   G       C       FAM138A G/G     G/G     G/G     G/G
chr1        30866   30869   CCT     C       FAM138A CCT/CCT CCT/CCT CCT/C   CCT/CCT
chr1        30894   30895   T       C       FAM138A T/C     T/C     T/T     T/T
chr1        30922   30923   G       T       FAM138A ./.     ./.     ./.     ./.
chr1        69269   69270   A       G       OR4F5   ./.     ./.     G/G     G/G
chr1        69427   69428   T       G       OR4F5   T/T     T/T     T/T     T/T
chr1        69510   69511   A       G       OR4F5   ./.     ./.     A/G     A/G
chr1        69760   69761   A       T       OR4F5   A/A     A/T     A/A     A/A
chr1        69870   69871   G       A       OR4F5   ./.     G/G     G/G     G/G
```

You can also add a header so that you can keep track of who's who:

```
$ gemini query -q "select chrom, start, end, ref, alt, gene, \
                              gts.1094PC0005, \
                              gts.1094PC0009, \
                              gts.1094PC0012, \
                              gts.1094PC0013 \
                       from variants" test.snpEff.vcf.db

chrom       start   end     ref     alt     gene gts.1094PC0005     gts.1094PC0009  gts.1094PC0012  g
chr1        30547   30548   T       G       FAM138A ./.     ./.     ./.     ./.
chr1        30859   30860   G       C       FAM138A G/G     G/G     G/G     G/G
chr1        30866   30869   CCT     C       FAM138A CCT/CCT CCT/CCT CCT/C   CCT/CCT
chr1        30894   30895   T       C       FAM138A T/C     T/C     T/T     T/T
chr1        30922   30923   G       T       FAM138A ./.     ./.     ./.     ./.
chr1        69269   69270   A       G       OR4F5   ./.     ./.     G/G     G/G
chr1        69427   69428   T       G       OR4F5   T/T     T/T     T/T     T/T
chr1        69510   69511   A       G       OR4F5   ./.     ./.     A/G     A/G
chr1        69760   69761   A       T       OR4F5   A/A     A/T     A/A     A/A
chr1        69870   69871   G       A       OR4F5   ./.     G/G     G/G     G/G
```

Let's now get the genotype and the depth of aligned sequence observed for a sample so that we can assess the confidence in the genotype:

```
$ gemini query -q "select chrom, start, end, ref, alt, gene,
                          gts.1094PC0005, \
                          gt_depths.1094PC0005, \
                   from variants" test.snpEff.vcf.db
```

```
chr1    30547   30548   T       G       FAM138A ./.     -1
chr1    30859   30860   G       C       FAM138A G/G     7
chr1    30866   30869   CCT     C       FAM138A CCT/CCT 8
chr1    30894   30895   T       C       FAM138A T/C     8
chr1    30922   30923   G       T       FAM138A ./.     -1
chr1    69269   69270   A       G       OR4F5   ./.     -1
chr1    69427   69428   T       G       OR4F5   T/T     2
chr1    69510   69511   A       G       OR4F5   ./.     -1
chr1    69760   69761   A       T       OR4F5   A/A     1
chr1    69870   69871   G       A       OR4F5   ./.     -1
```

2.5.3 Filtering on genotypes

Now, we often want to focus only on variants where a given sample has a specific genotype (e.g., looking for homozygous variants in family trios). Unfortunately, we cannot directly do this in the SQL query, but the *gemini query* tool has an option called *–gt-filter* that allows one to specify filters to apply to the returned rows. The rules followed in the *–gt-filter* option follow Python syntax.

Tip: As you will see from the examples below, appropriate use of the –gt-filter option will allow you to compose queries that return variants meeting inheritance patterns that are relevant to the disease model of interest in your study.

As an example, let's only return rows where sample 1094PC0012 is heterozygous. In order to do this, we apply a filter to the *gt_types* columns for this individual:

```
$ gemini query -q "select chrom, start, end, ref, alt, gene,
                      gts.1094PC0005, \
                      gts.1094PC0009, \
                      gts.1094PC0012, \
                      gts.1094PC0013 \
               from variants" \
               --gt-filter "gt_types.1094PC0012 == HET" \
               --header \
               test.snpEff.vcf.db

chrom   start   end     ref     alt     gene gts.1094PC0005     gts.1094PC0009  gts.1094PC0012  gts.1
chr1    30866   30869   CCT     C       FAM138A CCT/CCT CCT/CCT CCT/C   CCT/CCT
chr1    69510   69511   A       G       OR4F5   ./.     ./.     A/G     A/G
```

Now let's be a bit less restrictive and return variants where either sample 1094PC0012 is heterozygous or sample 1094PC0005 is homozygous for the reference allele:

```
$ gemini query -q "select chrom, start, end, ref, alt, gene,
                      gts.1094PC0005, \
                      gts.1094PC0009, \
                      gts.1094PC0012, \
                      gts.1094PC0013 \
               from variants" \
               --gt-filter "gt_types.1094PC0012 == HET or \
               gt_types.1094PC0005 == HOM_REF" \
               --header \
               test.snpEff.vcf.db

chrom   start   end     ref     alt     gene gts.1094PC0005     gts.1094PC0009  gts.1094PC0012  gts.1
chr1    30859   30860   G       C       FAM138A G/G     G/G     G/G     G/G
chr1    30866   30869   CCT     C       FAM138A CCT/CCT CCT/CCT CCT/C   CCT/CCT
chr1    69427   69428   T       G       OR4F5   T/T     T/T     T/T     T/T
```

```
chr1    69510   69511   A       G       OR4F5   ./.     ./.     A/G     A/G
chr1    69760   69761   A       T       OR4F5   A/A     A/T     A/A     A/A
```

Eh, I changed my mind, let's restrict the above to those variants where sample 1094PC0012 must also be heterozygous:

```
$ gemini query -q "select chrom, start, end, ref, alt, gene,
                      gts.1094PC0005, \
                      gts.1094PC0009, \
                      gts.1094PC0012, \
                      gts.1094PC0013 \
               from variants" \
               --gt-filter "(gt_types.1094PC0012 == HET or \
               gt_types.1094PC0005 == HOM_REF) \
               and \
               (gt_types.1094PC0013 == HET)" \
               --header \
               test.snpEff.vcf.db

 chrom   start   end     ref     alt     gene gts.1094PC0005     gts.1094PC0009  gts.1094PC0012  gts.1
 chr1    69510   69511   A       G       OR4F5   ./.     ./.     A/G     A/G
```

2.5.4 Finding out which samples have a variant

While exploring your data you might hit on a set of interesting variants and want to know which of your samples have that variant in them. You can display the samples containing a variant with the –show-sample-variants flag:

```
$ gemini query --header --show-samples -q "select chrom, start, end, ref, alt \
                                           from variants where is_lof=1 limit 5" test.query.db

chrom   start   end     ref     alt     variant_samples HET_samples     HOM_ALT_samples
chr1    874815  874816  C       CT      1478PC0006B,1478PC0007B,1478PC0010,1478PC0013B,1478PC0022B,14
chr1    1140811 1140813 TC      T       1478PC0011      1478PC0011
chr1    1219381 1219382 C       G       1719PC0012      1719PC0012
chr1    1221487 1221490 CAA     C       1478PC0004      1478PC0004
```

variant_samples is a list of all of the samples with a variant, HET_samples is the subset of those heterozygous for the variant and HOM_ALT_samples is the subset homozygous for the variant.

2.6 Built-in analysis tools

2.6.1 `comp_hets`: Identifying potential compound heterozygotes

Many recessive disorders are caused by compound heterozygotes. Unlike canonical recessive sites where the same recessive allele is inherited from both parents at the _same_ site in the gene, compound heterozygotes occur when the individual's phenotype is caused by two heterozygous recessive alleles at _different_ sites in a particular gene.

So basically, we are looking for two (typically loss-of-function (LoF)) heterozygous variants impacting the same gene at different loci. The complicating factor is that this is _recessive_ and as such, we must also require that the consequential alleles at each heterozygous site were inherited on different chromosomes (one from each parent). As such, in order to use this tool, we require that all variants are phased. Once this has been done, the *comp_hets* tool will provide a report of candidate compound heterozygotes for each sample/gene.

For example:

```
$ gemini comp_hets chr22.low.exome.snpeff.100samples.vcf.db
sample  gene    het1    het2
NA19675  PKDREJ  chr22,46653547,46653548,C,T,C|T,non_syn_coding,exon_22_46651560_46659219,0.005,1  ch
```

This indicates that sample NA19675 has a candidate compound heterozygote in PKDREJ. The two heterozygotes are reported using the following structure:

```
chrom,start,end,ref,alt,genotype,impact,exon,AAF,in_dbsnp
```

--only_lof

By default, all coding variants are explored. However, one may want to restrict the analysis to LoF variants using the `--only_lof` option.

```
$ gemini comp_hets --only_lof chr22.low.exome.snpeff.100samples.vcf.db
NA19002 GTSE1   chr22,46722400,46722401,G,A,G|A,stop_gain,exon_22,0.005,1       chr22,46704499,467045
```

--allow-other-hets

By default, the `comp_hets` tool will identify candidate pairs of heterozygotes that are found in *only one* of the samples in your database. Depending on the genetic model, this may be too restrictive. If you'd like to identify candidates where other individuals may also be heterozygous, just use the `--allow-other-hets` option

```
$ gemini comp_hets --allow-other-hets chr22.low.exome.snpeff.100samples.vcf.db
NA19375  PKDREJ  chr22,46658977,46658978,T,C,T|C,non_syn_coding,exon_22_46651560_46659219,0.25,1  chr
HG01619  PKDREJ  chr22,46658977,46658978,T,C,C|T,non_syn_coding,exon_22_46651560_46659219,0.25,1  chr
```

Here, samples NA19375 and HG01619 are both hets for the same variant (chr22,46658977,46658978)

--ignore-phasing

If your genotypes aren't phased, we can't be certain that two heterozygotes are on opposite alleles. However, we can still identify pairs of heterozygotes that are *candidates* for compound heterozygotes. Just use the `--ignore-phasing` option.

```
$ gemini comp_hets --ignore_phasing example.db
M1047  DHODH  chr16,72048539,72048540,C,T,C/T,non_syn_coding,3/4,0.125,1  chr16,72057434,72057435,C,T
M1282  DHODH  chr16,72055099,72055100,C,T,C/T,non_syn_coding,5/9,0.125,0  chr16,72055114,72055116,CT,
```

2.6.2 `de_novo`: Identifying potential de novo mutations.

Note: This tool requires that you identify familial relationships via a PED file when loading your VCF into gemini via:

```
gemini load -v my.vcf -p my.ped my.db
```

Example PED file format for GEMINI

```
#Family_ID      Individual_ID   Paternal_ID     Maternal_ID     Sex     Phenotype       Ethnicity
1       S173    S238    S239    1       2       caucasian
1       S238    -9      -9      1       1       caucasian
1       S239    -9      -9      2       1       caucasian
```

```
2       S193    S230    S231    1       2       caucasian
2       S230    -9      -9      1       1       caucasian
2       S231    -9      -9      2       1       caucasian
3       S242    S243    S244    1       2       caucasian
3       S243    -9      -9      1       1       caucasian
3       S244    -9      -9      2       1       caucasian
4       S253    S254    S255    1       2       caucasianNEuropean
4       S254    -9      -9      1       1       caucasianNEuropean
4       S255    -9      -9      2       1       caucasianNEuropean
```

Assuming you have defined the familial relationships between samples when loading your VCF into GEMINI, one can leverage a built-in tool for identifying de novo (a.k.a spontaneous) mutations that arise in offspring.

`default behavior`

By default, the `de novo` tool will report, for each family in the database, a list of mutations that are not found in the parents yet are observed as heterozygotes in the offspring. For example:

```
$ gemini de_novo my.db

family_id       chrom   start   end     ref     alt     gene    impact  impact_severity in_dbsnp
1       chr1    17197609        17197610        G       A       BX284668.1      non_syn_coding  MED
1       chr1    196763706       196763707       T       C       CFHR3   splice_acceptor HIGH    1
1       chr1    248813541       248813542       G       A       OR2T27  non_syn_coding  MED     1
1       chr2    90060872        90060873        A       T       AC009958.1      non_syn_coding  MED
1       chr3    195505789       195505790       G       C       MUC4    non_syn_coding  MED     1
...
```

`-d`

Unfortunately, inherited variants can often appear to be de novo mutations simply because insufficient sequence coverage was available for one of the parents to detect that the parent(s) is also a heterozygote (and thus the variant was actually inherited, not spontaneous). One simple way to filter such artifacts is to enforce a minimum sequence depth for each sample. For example, if we require that at least 50 sequence alignments were present for mom, dad and child, two of the above variants will be eliminated as candidates:

```
$ gemini de_novo -d 50 my.db

family_id       chrom   start   end     ref     alt     gene    impact  impact_severity in_dbsnp
1       chr1    17197609        17197610        G       A       BX284668.1      non_syn_coding  MED
1       chr2    90060872        90060873        A       T       AC009958.1      non_syn_coding  MED
1       chr3    195505789       195505790       G       C       MUC4    non_syn_coding  MED     1
...
```

2.6.3 `autosomal_recessive`: Find variants meeting an autosomal recessive model.

Note: This tool requires that you identify familial relationships via a PED file when loading your VCF into gemini via:

```
gemini load -v my.vcf -p my.ped my.db
```

Assuming you have defined the familial relationships between samples when loading your VCF into GEMINI, one can leverage a built-in tool for identifying variants that meet an autosomal recessive inheritance pattern. The reported variants will be restricted to those variants having the potential to impact the function of affecting protein coding transcripts.

```
$ gemini autosomal_recessive my.db | head

family_id       chrom   start   end     ref     alt     gene    impact  impact_severity sample1(fathe
1       chr1    1888192 1888193 C       A       C1orf222        non_syn_coding  MED     C/A     C/A
1       chr1    6162053 6162054 T       C       CHD5    non_syn_coding  MED     T/C     T/C     C/C
1       chr1    6646958 6646968 GCCTGCCTTC      G       ZBTB48  inframe_codon_loss      MED     GCCTC
1       chr1    11826629        11826630        C       T       C1orf167        non_syn_coding  MED
1       chr1    11828237        11828238        G       A       C1orf167        non_syn_coding  MED
1       chr1    11828318        11828319        G       A       C1orf167        non_syn_coding  MED
1       chr1    11831614        11831615        C       T       C1orf167        non_syn_coding  MED
1       chr1    11836627        11836628        T       C       C1orf167        non_syn_coding  MED
1       chr1    11836681        11836682        C       T       C1orf167        non_syn_coding  MED
```

2.6.4 `autosomal_dominant`: Find variants meeting an autosomal dominant model.

Note: This tool requires that you identify familial relationships via a PED file when loading your VCF into gemini via:

```
gemini load -v my.vcf -p my.ped my.db
```

Assuming you have defined the familial relationships between samples when loading your VCF into GEMINI, one can leverage a built-in tool for identifying variants that meet an autosomal dominant inheritance pattern. The reported variants will be restricted to those variants having the potential to impact the function of affecting protein coding transcripts.

```
$ gemini autosomal_dominant my.db | head

family_id       chrom   start   end     ref     alt     gene    impact  impact_severity sample1(fathe
1       chr1    16855   16856   A       G       WASH7P  splice_donor    HIGH    A/A     A/G     A/G
1       chr1    881917  881918  G       A       NOC2L   non_syn_coding  MED     G/A     G/G     G/A
1       chr1    907757  907758  A       G       PLEKHN1 non_syn_coding  MED     A/A     A/G     A/G
1       chr1    909237  909238  G       C       PLEKHN1 non_syn_coding  MED     G/C     C/C     G/C
1       chr1    916548  916549  A       G       C1orf170        non_syn_coding  MED     A/G     G/G
1       chr1    935221  935222  C       A       HES4    non_syn_coding  MED     C/A     A/A     C/A
1       chr1    949607  949608  G       A       ISG15   non_syn_coding  MED     G/A     G/G     G/A
1       chr1    979747  979748  A       T       AGRN    non_syn_coding  MED     A/T     A/A     A/T
1       chr1    1361529 1361530 C       T       TMEM88B non_syn_coding  MED     C/T     C/C     C/T
```

2.6.5 `pathways`: Map genes and variants to KEGG pathways.

Mapping genes to biological pathways is useful in understanding the function/role played by a gene. Likewise, genes involved in common pathways is helpful in understanding heterogeneous diseases. We have integrated the KEGG pathway mapping for gene variants, to explain/annotate variation. This requires your VCF be annotated with either snpEff/VEP.

Examples:

```
$ gemini pathways -v 68 example.db
chrom   start   end     ref     alt     impact  sample  genotype        gene    transcript      pathw
```

```
chr10   52004314        52004315        T       C       intron  M128215 C/C     ASAH2   ENST00000395
chr10   126678091       126678092       G       A       stop_gain       M128215 G/A     CTBP2   ENST
chr16   72057434        72057435        C       T       non_syn_coding  M10475  C/T     DHODH   ENST
```

Here, -v specifies the version of the Ensembl genes used to build the KEGG pathway map. Hence, use versions that match the VEP/snpEff versions of the annotated vcf for correctness. For e.g VEP v2.6 and snpEff v3.1 use Ensembl 68 version of the genomes.

We currently support versions 66 through 71 of the Ensembl genes

--lof

By default, all gene variants that map to pathways are reported. However, one may want to restrict the analysis to LoF variants using the `--lof` option.

```
$ gemini pathways --lof -v 68 example.db
chrom   start   end     ref     alt     impact  sample  genotype        gene    transcript      path
chr10   126678091       126678092       G       A       stop_gain       M128215 G/A     CTBP2   ENST
```

2.6.6 `interactions`: Find genes among variants that are interacting partners.

Integrating the knowledge of the known protein-protein interactions would be useful in explaining variation data. Meaning to say that a damaging variant in an interacting partner of a potential protein may be equally interesting as the protein itself. We have used the HPRD binary interaction data to build a p-p network graph which can be explored by Gemini.

Examples:

```
$ gemini interactions -g CTBP2 -r 3 example.db
sample  gene    order_of_interaction    interacting_gene
M128215 CTBP2   0_order:        CTBP2
M128215 CTBP2   1_order:        RAI2
M128215 CTBP2   2_order:        RB1
M128215 CTBP2   3_order:        TGM2,NOTCH2NL
```

Return CTBP2 (-g) interacting gene variants till the third order (-r)

lof_interactions

Use this option to restrict your analysis to only LoF variants.

```
$ gemini lof_interactions -r 3 example.db
sample  lof_gene        order_of_interaction    interacting_gene
M128215 TGM2    1_order:        RB1
M128215 TGM2    2_order:        none
M128215 TGM2    3_order:        NOTCH2NL,CTBP2
```

Meaning to say return all LoF gene TGM2 (in sample M128215) interacting partners to a 3rd order of interaction.

--var

An extended variant information (chrom, start, end etc.) for the interacting gene may be achieved with the –var option for both the `interactions` and the `lof_interactions`

```
$ gemini interactions -g CTBP2 -r 3 --var example.db
sample  gene    order_of_interaction    interacting_gene        var_id  chrom   start   end     impac
M128215 CTBP2   0       CTBP2   5       chr10   126678091       126678092       stop_gain       prote
M128215 CTBP2   1       RAI2    9       chrX    17819376        17819377        non_syn_coding  prote
M128215 CTBP2   2       RB1     7       chr13   48873834        48873835        upstream        prote
M128215 CTBP2   3       NOTCH2NL        1       chr1    145273344       145273345       non_syn_codir
M128215 CTBP2   3       TGM2    8       chr20   36779423        36779424        stop_gain       prote

$ gemini lof_interactions -r 3 --var example.db
sample  lof_gene        order_of_interaction    interacting_gene        var_id  chrom   start   end
M128215 TGM2    1       RB1     7       chr13   48873834        48873835        upstream        prote
M128215 TGM2    3       NOTCH2NL        1       chr1    145273344       145273345       non_syn_codir
M128215 TGM2    3       CTBP2   5       chr10   126678091       126678092       stop_gain       prote
```

2.6.7 `lof_sieve`: Filter LoF variants by transcript position and type

Not all candidate LoF variants are created equal. For e.g, a nonsense (stop gain) variant impacting the first 5% of a polypeptide is far more likely to be deleterious than one affecting the last 5%. Assuming you've annotated your VCF with snpEff v3.0+, the lof_sieve tool reports the fractional position (e.g. 0.05 for the first 5%) of the mutation in the amino acid sequence. In addition, it also reports the predicted function of the transcript so that one can segregate candidate LoF variants that affect protein_coding transcripts from processed RNA, etc.

```
$ gemini lof_sieve chr22.low.exome.snpeff.100samples.vcf.db
chrom   start   end ref alt highest_impact  aa_change   var_trans_pos   trans_aa_length var_trans_pct
chr22   17072346    17072347    C   T   stop_gain   W365*   365 557 0.655296229803  NA19327 C|T CCT8I
chr22   17072346    17072347    C   T   stop_gain   W365*   365 557 0.655296229803  NA19375 T|C CCT8I
chr22   17129539    17129540    C   T   splice_donor    None    None    None    None    NA18964 T|C T
chr22   17129539    17129540    C   T   splice_donor    None    None    None    None    NA19675 T|C T
```

2.6.8 `annotate`: adding your own custom annotations

It is inevitable that researchers will want to enhance the gemini framework with their own, custom annotations. `gemini` provides a sub-command called `annotate` for exactly this purpose. As long as you provide a `tabix`'ed annotation file in either BED or VCF format, the annotate tool will, for each variant in the variants table, screen for overlaps in your annotation file and update a new column in the variants table that you may specify on the command line. This is best illustrated by example.

Let's assume you have already created a gemini database of a VCF file using the `load` module.

```
$ gemini load -v my.vcf -t snpEff my.db
```

Now, let's imagine you have an annotated file in BED format (`crucial.bed`) that describes regions of the genome that are particularly relevant to your lab's research. You would like to annotate in the gemini database which variants overlap these crucial regions. We want to store this knowledge in a new column in the `variants` table called `crucial_variant` that tracks whether a given variant overlapped (1) or did not overlap (0) intervals in your annotation file.

To do this, you must first TABIX your BED file:

```
$ bgzip crucial.bed
$ tabix -p bed crucial.bed.gz
```

`-t boolean` Did a variant overlap a region or not?

Now, you can use this TABIX'ed file to annotate which variants overlap your crucial regions. In the example below, the results will be stored in a new column called "crucial". The `-t boolean` option says that you just want to track whether (1) or not (0) the variant overlapped one or more of your regions.

```
$ gemini annotate -f crucial.bed.gz -c crucial -t boolean my.db
```

Since a new columns has been created in the database, we can now directly query the new column. In the example results below, the first and third variants overlapped a crucial region while the second did not.

```
$ gemini query \
    -q "select chrom, start, end, variant_id, crucial from variants" \
    my.db \
    | head -3
chr22   100     101     1    1
chr22   200     201     2    0
chr22   300     500     3    1
```

`-t count` How many regions did a variant overlap?

Instead of a simple yes or no, we can use the `-t count` option to *count* how many crucial regions a variant overlapped. It turns out that the 3rd variant actually overlapped two crucial regions.

```
$ gemini annotate -f crucial.bed.gz -c crucial -t count my.db

$ gemini query \
    -q "select chrom, start, end, variant_id, crucial from variants" \
    my.db \
    | head -3
chr22   100     101     1    1
chr22   200     201     2    0
chr22   300     500     3    2
```

`-t list` Which regions did a variant overlap?

Lastly, we can *list* which regions a variant overlapped using the `-t list` option. Let's imaging that `crucial.bed` looks like this:

```
chr22   50    150    crucial1
chr22   300   400    crucial2
chr22   350   450    crucial3
```

When we use `-t list`, the resulting column can store a comma-separated list of the region names (column 4). You can choose whatever column you want to store in the database, but in this example, we will use the 4th column (the name). We specify which column to store in the list with the `-e` option.

```
$ gemini annotate -f crucial.bed.gz -c crucial -t list -e 4 my.db

$ gemini query \
    -q "select chrom, start, end, variant_id, crucial from variants" \
    my.db \
    | head -3
chr22   100     101     1    crucial1
chr22   200     201     2    0
chr22   300     500     3    crucial2,crucial3
```

2.6.9 `region`: Extracting variants from specific regions or genes

One often is concerned with variants found solely in a particular gene or genomic region. `gemini` allows one to extract variants that fall within specific genomic coordinates as follows:

`--reg`

```
$ gemini region --reg chr1:100-200 my.db
```

`--gene`

Or, one can extract variants based on a specific gene name.

```
$ gemini region --gene PTPN22 my.db
```

2.6.10 `windower`: Conducting analyses on genome "windows".

`gemini` includes a convenient tool for computing variation metrics across genomic windows (both fixed and sliding). Here are a few examples to whet your appetite. If you're still hungry, contact us.

Compute the average nucleotide diversity for all variants found in non-overlapping, 50Kb windows.

```
$ gemini windower -w 50000 -s 0 -t nucl_div -o mean my.db
```

Compute the average nucleotide diversity for all variants found in 50Kb windows that overlap by 10kb.

```
$ gemini windower -w 50000 -s 10000 -t nucl_div -o mean my.db
```

Compute the max value for HWE statistic for all variants in a window of size 10kb

```
$ gemini windower  -w 10000 -t hwe -o max my.db
```

2.6.11 `stats`: Compute useful variant statistics.

The `stats` tool computes some useful variant statistics like

Compute the transition and transversion ratios for the snps

```
$ gemini stats --tstv my.db
ts      tv      ts/tv
4       5       0.8
```

`--tstv-coding`

Compute the transition/transversion ratios for the snps in the coding regions.

`--tstv-noncoding`

Compute the transition/transversion ratios for the snps in the non-coding regions.

Compute the type and count of the snps.

```
$ gemini stats --snp-counts my.db
type    count
A->G    2
C->T    1
G->A    1
```

Calculate the site frequency spectrum of the variants.

```
$ gemini stats --sfs my.db
aaf     count
0.125   2
0.375   1
```

Compute the pair-wise genetic distance between each sample

```
$ gemini stats --mds my.db
sample1 sample2 distance
M10500  M10500  0.0
M10475  M10478  1.25
M10500  M10475  2.0
M10500  M10478  0.5714
```

Return a count of the types of genotypes per sample

```
$ gemini stats --gts-by-sample my.db
sample  num_hom_ref     num_het num_hom_alt     num_unknown     total
M10475  4       1       3       1       9
M10478  2       2       4       1       9
```

Return the total variants per sample (sum of homozygous and heterozygous variants)

```
$ gemini stats --vars-by-sample my.db
sample  total
M10475  4
M10478  6
```

--summarize

If none of these tools are exactly what you want, you can summarize the variants per sample of an arbitrary query using the –summarize flag. For example, if you wanted to know, for each sample, how many variants are on chromosome 1 that are also in dbSNP:

```
$ gemini stats --summarize "select * from variants where in_dbsnp=1 and chrom='chr1'" my.db
sample  total   num_het num_hom_alt
M10475  1       1       0
M128215 1       1       0
M10478  2       2       0
M10500  2       1       1
```

2.6.12 `db_info`: List the gemini database tables and columns

Because of the sheer number of annotations that are stored in gemini, there are admittedly too many columns to remember by rote. If you can recall the name of particular column, just use the `db_info` tool. It will report all of the tables and all of the columns / types in each table:

```
$ gemini db_info test.db
table_name          column_name                    type
variants            chrom                          text
variants            start                          integer
variants            end                            integer
variants            variant_id                     integer
variants            anno_id                        integer
variants            ref                            text
variants            alt                            text
variants            qual                           float
variants            filter                         text
variants            type                           text
variants            sub_type                       text
variants            gts                            blob
variants            gt_types                       blob
variants            gt_phases                      blob
variants            gt_depths                      blob
variants            call_rate                      float
variants            in_dbsnp                       bool
variants            rs_ids                         text
variants            in_omim                        bool
variants            clin_sigs                      text
variants            cyto_band                      text
variants            rmsk                           text
variants            in_cpg_island                  bool
variants            in_segdup                      bool
variants            is_conserved                   bool
variants            num_hom_ref                    integer
variants            num_het                        integer
variants            num_hom_alt                    integer
variants            num_unknown                    integer
variants            aaf                            float
variants            hwe                            float
variants            inbreeding_coeff               float
variants            pi                             float
variants            recomb_rate                    float
variants            gene                           text
variants            transcript                     text
variants            is_exonic                      bool
variants            is_coding                      bool
variants            is_lof                         bool
variants            exon                           text
variants            codon_change                   text
variants            aa_change                      text
variants            aa_length                      text
variants            biotype                        text
variants            impact                         text
variants            impact_severity                text
variants            polyphen_pred                  text
variants            polyphen_score                 float
variants            sift_pred                      text
variants            sift_score                     float
variants            anc_allele                     text
variants            rms_bq                         float
variants            cigar                          text
variants            depth                          integer
variants            strand_bias                    float
variants            rms_map_qual                   float
```

```
variants            in_hom_run                    integer
variants            num_mapq_zero                 integer
variants            num_alleles                   integer
variants            num_reads_w_dels              float
variants            haplotype_score               float
variants            qual_depth                    float
variants            allele_count                  integer
variants            allele_bal                    float
variants            in_hm2                        bool
variants            in_hm3                        bool
variants            is_somatic
variants            in_esp                        bool
variants            aaf_esp_ea                    float
variants            aaf_esp_aa                    float
variants            aaf_esp_all                   float
variants            exome_chip                    bool
variants            in_1kg                        bool
variants            aaf_1kg_amr                   float
variants            aaf_1kg_asn                   float
variants            aaf_1kg_afr                   float
variants            aaf_1kg_eur                   float
variants            aaf_1kg_all                   float
variants            grc                           text
variants            gms_illumina                  float
variants            gms_solid                     float
variants            gms_iontorrent                float
variants            encode_tfbs
variants            encode_consensus_gm12878      text
variants            encode_consensus_h1hesc       text
variants            encode_consensus_helas3       text
variants            encode_consensus_hepg2        text
variants            encode_consensus_huvec        text
variants            encode_consensus_k562         text
variants            encode_segway_gm12878         text
variants            encode_segway_h1hesc          text
variants            encode_segway_helas3          text
variants            encode_segway_hepg2           text
variants            encode_segway_huvec           text
variants            encode_segway_k562            text
variants            encode_chromhmm_gm12878       text
variants            encode_chromhmm_h1hesc        text
variants            encode_chromhmm_helas3        text
variants            encode_chromhmm_hepg2         text
variants            encode_chromhmm_huvec         text
variants            encode_chromhmm_k562          text
variant_impacts     variant_id                    integer
variant_impacts     anno_id                       integer
variant_impacts     gene                          text
variant_impacts     transcript                    text
variant_impacts     is_exonic                     bool
variant_impacts     is_coding                     bool
variant_impacts     is_lof                        bool
variant_impacts     exon                          text
variant_impacts     codon_change                  text
variant_impacts     aa_change                     text
variant_impacts     aa_length                     text
variant_impacts     biotype                       text
variant_impacts     impact                        text
```

```
variant_impacts     impact_severity           text
variant_impacts     polyphen_pred             text
variant_impacts     polyphen_score            float
variant_impacts     sift_pred                 text
variant_impacts     sift_score                float
samples             sample_id                 integer
samples             name                      text
samples             family_id                 integer
samples             paternal_id               integer
samples             maternal_id               integer
samples             sex                       text
samples             phenotype                 text
samples             ethnicity                 text
```

2.7 The GEMINI browser interface

Currently, the majority of GEMINI's functionality is available via a command-line interface. However, we are developing a browser-based interface for easier exploration of GEMINI databases created with the `gemini load` command.

Ironically, as of now, one must launch said browser from the command line as follows (where `my.db` should be replaced with the name of the GEMINI database you would like to explore).

```
$ gemini browser my.db
```

At this point, the GEMINI browser is running on port 8088 on your local machine. Open a web browser to http://localhost:8088/query You should see something like:

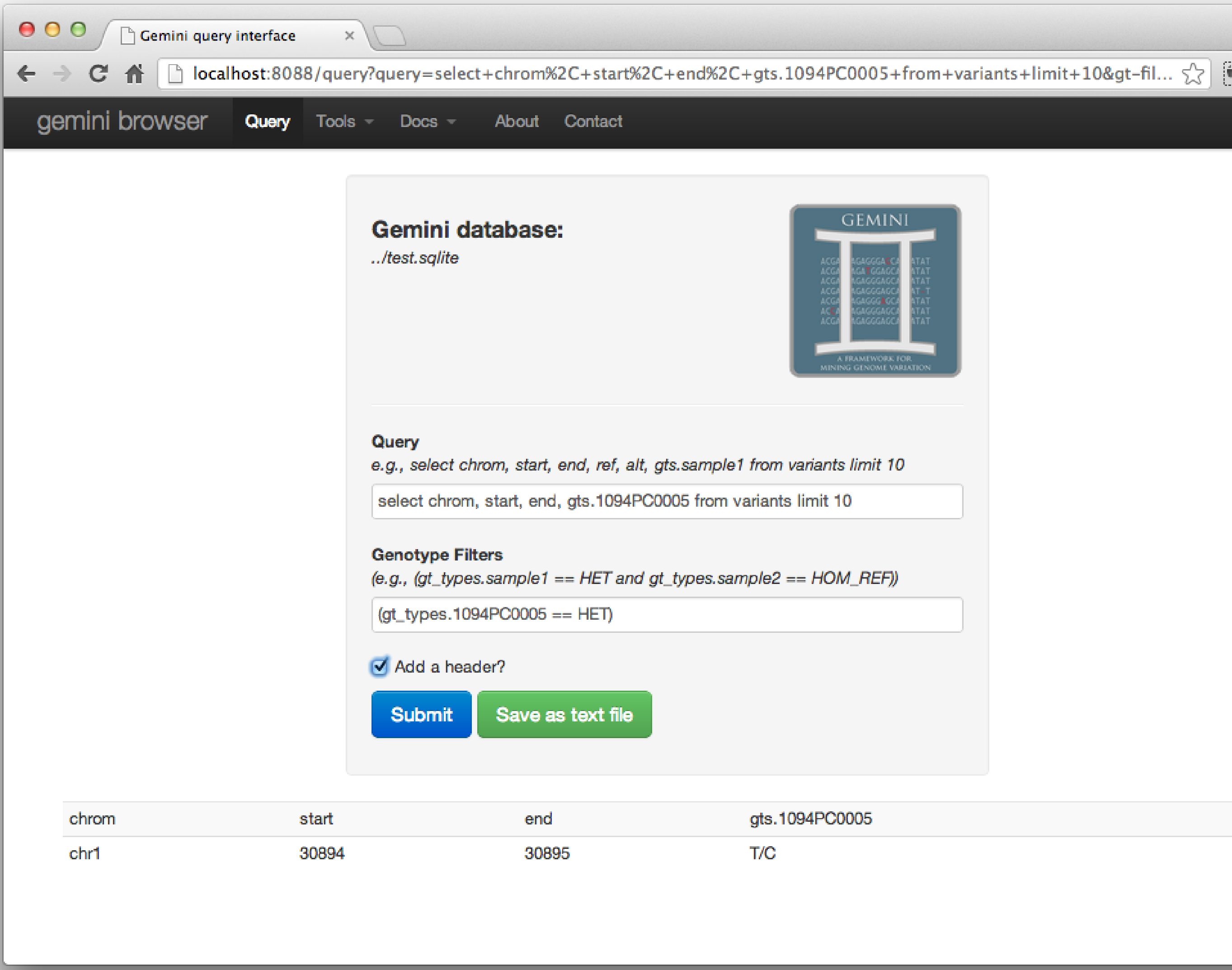
Gemini query interface
localhost:8088/query?query=select+chrom%2C+start%2C+end%2C+gts.1094PC0005+from+variants+limit+10&gt-fil...
gemini browser
Query
Tools
Docs
About
Contact
Gemini database:
../test.sqlite
GEMINI
A FRAMEWORK FOR MINING GENOME VARIATION
Query
e.g., select chrom, start, end, ref, alt, gts.sample1 from variants limit 10
select chrom, start, end, gts.1094PC0005 from variants limit 10
Genotype Filters
(e.g., (gt_types.sample1 == HET and gt_types.sample2 == HOM_REF))
(gt_types.1094PC0005 == HET)
Add a header?
Submit
Save as text file
chrom
start
end
gts.1094PC0005
chr1
30894
30895
T/C

2.8 The Gemini database schema

2.8.1 The `variants` table

Core VCF fields

column_name	type	notes
chrom	STRING	The chromosome on which the variant resides
start	INTEGER	The 0-based start position.
end	INTEGER	The 1-based end position.
variant_id	INTEGER	PRIMARY_KEY
anno_id	INTEGER	Variant transcript number for the most severely affected transcript
ref	STRING	Reference allele
alt	STRING	Alternate alele for the variant
qual	INTEGER	Quality score for the assertion made in ALT
filter	STRING	A string of filters passed/failed in variant calling

Variant and PopGen info

type	STRING	The type of variant.<br>Any of: [*snp*, *indel*]
sub_type	STRING	The variant sub-type.<br>If `type` is *snp*: [*ts*, (transition), *tv* (transversion)]<br>If `type` is *indel*: [*ins*, (insertion), *del* (deletion)]
call_rate	FLOAT	The fraction of samples with a valid genotype
num_hom_ref	INTEGER	The total number of of homozygotes for the reference (`ref`) allele
num_het	INTEGER	The total number of heterozygotes observed.
num_hom_alt	INTEGER	The total number of homozygotes for the reference (`alt`) allele
num_unknown	INTEGER	The total number of of unknown genotypes
aaf	FLOAT	The observed allele frequency for the alternate allele
hwe	FLOAT	The Chi-square probability of deviation from HWE (assumes random mating)
inbreeding_coeff	FLOAT	The inbreeding co-efficient that expresses the likelihood of effects due to inbreeding
pi	FLOAT	The computed nucleotide diversity (pi) for the site

Genotype information

gts	BLOB	A compressed binary vector of sample genotypes (e.g., “A/A”, “A|G”, “G/G”)
gt_types	BLOB	A compressed binary vector of numeric genotype “types” (e.g., 0, 1, 2)
gt_phases	BLOB	A compressed binary vector of sample genotype phases (e.g., False, True, False)
gt_depths	BLOB	A compressed binary vector of the depth of aligned sequence observed for each sample

Gene information

gene	STRING	Corresponding gene name of the highly affected transcript
transcript	STRING	The variant transcript that was most severely affected<br>(for two equally affected transcripts, either the first one is selected (VEP) or the protein_coding biotype is prioritized (snpEff)
is_exonic	BOOL	Does the variant affect an exon for >= 1transcript?
is_coding	BOOL	Does the variant fall in a coding region (excl. 3' & 5' UTRs) for >= 1 transcript?
is_lof	BOOL	Based on the value of the impact col, is the variant LOF for >= transcript?
exon	STRING	Exon information for the severely affected transcript
codon_change	STRING	What is the codon change?
aa_change	STRING	What is the amino acid change (for an snp)?
aa_length	STRING	The length of CDS in terms of number of amino acids (`only snpEff`)
biotype	STRING	The 'type' of the severely affected transcript (e.g.protein-coding, pseudogene, rRNA etc.) (`only snpEff`)
impact	STRING	The consequence of the most severely affected transcript
impact_severity	STRING	Severity of the highest order observed for the variant
polyphen_pred	STRING	Polyphen predictions for the snps for the severely affected transcript (`only VEP`)
polyphen_score	FLOAT	Polyphen scores for the severely affected transcript (`only VEP`)
sift_pred	STRING	SIFT predictions for the snp's for the most severely affected transcript (`only VEP`)
sift_score	FLOAT	SIFT scores for the predictions (`only VEP`)
pfam_domain	STRING	Pfam protein domain that the variant affects

Optional VCF INFO fields

anc_allele	STRING	The reported ancestral allele if there is one.
rms_bq	FLOAT	The RMS base quality at this position.
cigar	STRING	CIGAR string describing how to align an alternate allele to the reference allele.
depth	INTEGER	The number of aligned sequence reads that led to this variant call
strand_bias	FLOAT	Strand bias at the variant position
rms_map_qual	FLOAT	RMS mapping quality, a measure of variance of quality scores
in_hom_run	INTEGER	Homopolymer runs for the variant allele
num_mapq_zero	INTEGER	Total counts of reads with mapping quality equal to zero
num_alleles	INTEGER	Total number of alleles in called genotypes
num_reads_w_dels	FLOAT	Fraction of reads with spanning deletions
haplotype_score	FLOAT	Consistency of the site with two segregating haplotypes
qual_depth	FLOAT	Variant confidence or quality by depth
allele_count	INTEGER	Allele counts in genotypes
allele_bal	FLOAT	Allele balance for hets
is_somatic	BOOL	Whether the variant is somatically acquired.

Population information

in_dbsnp	BOOL	Is this variant found in dbSnp (build 135)?<br>0 : Absence of the variant in dbsnp<br>1 : Presence of the variant in dbsnp
rs_ids	STRING	A comma-separated list of rs ids for variants present in dbsnp
in_hm2	BOOL	Whether the variant was part of HapMap2.
in_hm3	BOOL	Whether the variant was part of HapMap3.
in_esp	BOOL	Presence/absence of the variant in the ESP project data
in_1kg	BOOL	Presence/absence of the variant in the 1000 genome project data
aaf_esp_ea	FLOAT	Minor Allele Frequency of the variant for European Americans in the ESP project
aaf_esp_aa	FLOAT	Minor Allele Frequency of the variant for African Americans in the ESP project
aaf_esp_all	FLOAT	Minor Allele Frequency of the variant w.r.t both groups in the ESP project
aaf_1kg_amr	FLOAT	Allele Frequency of the variant for samples in AMR based on AC/AN (1000g project)
aaf_1kg_asn	FLOAT	Allele frequency of the variant for samples in ASN based on AC/AN (1000g project)
aaf_1kg_afr	FLOAT	Allele frequency of the variant for samples in AFR based on AC/AN (1000g project)
aaf_1kg_eur	FLOAT	Allele Frequency of the variant for samples in EUR based on AC/AN (1000g project)
aaf_1kg_all	FLOAT	Global allele frequency (based on AC/AN) (1000g project)

Disease phenotype info (from ClinVar).

in_omim	BOOL	0 : Absence of the variant in OMIM database<br>1 : Presence of the variant in OMIM database
clinvar_sig	STRING	The clinical significance scores for each<br>of the variant according to ClinVar:<br>*unknown*, *untested*, *non-pathogenic*<br>*probable-non-pathogenic*, *probable-pathogenic*<br>*pathogenic*, *drug-response*, *histocompatibility*<br>*other*
clinvar_disease_name	STRING	The name of the disease to which the variant is relevant
clinvar_dbsource	STRING	Variant Clinical Channel IDs
clinvar_dbsource_id	STRING	The record id in the above database
clinvar_origin	STRING	The type of variant.<br>Any of:<br>*unknown*, *germline*, *somatic*,<br>*inherited*, *paternal*, *maternal*,<br>*de-novo*, *biparental*, *uniparental*,<br>*not-tested*, *tested-inconclusive*,<br>*other*
clinvar_dsdb	STRING	Variant disease database name
clinvar_dsdbid	STRING	Variant disease database ID
clinvar_disease_acc	STRING	Variant Accession and Versions
clinvar_in_locus_spec_db	BOOL	Submitted from a locus-specific database?
clinvar_on_diag_assay	BOOL	Variation is interrogated in a clinical diagnostic assay?

Genome annotations

exome_chip	BOOL	Whether an SNP is on the Illumina HumanExome Chip
cyto_band	STRING	Chromosomal cytobands that a variant overlaps
rmsk	STRING	A comma-separated list of RepeatMasker annotations that the variant overlaps.<br>Each hit is of the form: `name_class_family`
in_cpg_island	BOOL	Does the variant overlap a CpG island?.<br>Based on UCSC: Regulation > CpG Islands > cpgIslandExt
in_segdup	BOOL	Does the variant overlap a segmental duplication?.<br>Based on UCSC: Variation&Repeats > Segmental Dups > genomicSuperDups track
is_conserved	BOOL	Does the variant overlap a conserved region?<br>Based on the 29-way mammalian conservation study
gerp_bp_score	FLOAT	GERP conservation score.<br>Only populated if the `--load-gerp-bp` option is used when loading.<br>Higher scores reflect greater conservation. **At base-pair resolution**.<br>Details: http://mendel.stanford.edu/SidowLab/downloads/gerp
gerp_element_pval	FLOAT	GERP elements P-val<br>Lower P-values scores reflect greater conservation. **Not at base-pair resolution**.<br>Details: http://mendel.stanford.edu/SidowLab/downloads/gerp
recomb_rate	FLOAT	Returns the mean recombination rate [illegible]

Variant error assessment

grc	STRING	Association with patch and fix regions from the Genome Reference Consortium:<br>http://www.ncbi.nlm.nih.gov/projects/genome/assem[illegible]<br>Identifies potential problem regions associated with variant calls.<br>Built with *annotation_provenance/make-ncbi-grc-patches.py*
gms_illumina	FLOAT	Genome Mappability Scores (GMS) for Illumina error models<br>Provides low GMS scores (< 25.0 in any technology) from:<br>http://sourceforge.net/apps/mediawiki/gma-bio/index.php?title=Download_GMS<br>#Download_GMS_by_Chromosome_and_Sequencin[illegible]<br>Input VCF for annotations prepared with:<br>https://github.com/chapmanb/bcbio.variation/blob/ma[illegible]
gms_solid	FLOAT	Genome Mappability Scores with SOLiD error models
gms_iontorrent	FLOAT	Genome Mappability Scores with IonTorrent error models
in_cse	BOOL	Is a variant in an error prone genomic position,<br>using CSE: Context-Specific Sequencing Errors<br>https://code.google.com/p/discovering-cse/<br>http://www.biomedcentral.com/1471-2105/14/S5/S1

ENCODE information

encode_tfbs	STRING	Comma-separated list of transcription factors that were observed by ENCODE to bind DNA in this region. Each hit in the list is constructed<br>as TF_CELLCOUNT, where:<br>*TF* is the transcription factor name<br>*CELLCOUNT* is the number of cells tested that had nonzero signals.<br>Provenance: wgEncodeRegTfbsClusteredV2 UCSC table
encode_dnaseI_cell_count	INTEGER	Count of cell types that were observed to have DnaseI hypersensitivity.
encode_dnaseI_cell_list	STRING	Comma separated list of cell types that were observed to have DnaseI hypersensitivity.<br>Provenance: Thurman, et al, *Nature*, 489, pp. 75-82, 5 Sep. 2012
encode_consensus_gm12878	STRING	ENCODE consensus segmentation prediction for GM12878.<br><br>CTCF: CTCF-enriched element<br>E: Predicted enhancer<br>PF: Predicted promoter flanking region<br>R: Predicted repressed or low-activity region<br>TSS: Predicted promoter region including TSS<br>T: Predicted transcribed region<br>WE: Predicted weak enhancer or open chromatin cis-regulatory element | unknown: This region of the genome had no functional prediction.
encode_consensus_h1hesc	STRING	ENCODE consensus segmentation prediction for h1HESC. See encode_consseg_gm12878 for details.
encode_consensus_helas3	STRING	ENCODE consensus segmentation prediction for Helas3. See en-[illegible]

2.8.2 The `variant_impacts` table

column_name	type	notes
variant_id	INTEGER	PRIMARY_KEY (Foreign key to *variants* table)
anno_id	INTEGER	PRIMARY_KEY (Based on variant transcripts)
gene	STRING	The gene affected by the variant.
transcript	STRING	The transcript affected by the variant.
is_exonic	BOOL	Does the variant affect an exon for this transcript?
is_coding	BOOL	Does the variant fall in a coding region (excludes 3' & 5' UTR's of exons)?
is_lof	BOOL	Based on the value of the impact col, is the variant LOF?
exon	STRING	Exon information for the variants that are exonic
codon_change	STRING	What is the codon change?
aa_change	STRING	What is the amino acid change?
aa_length	STRING	The length of CDS in terms of number of amino acids (`snpEff only`)
biotype	STRING	The type of transcript (e.g.protein-coding, pseudogene, rRNA etc.) (`SnpEff only`)
impact	STRING	Impacts due to variation (ref.impact category)
impact_severity	STRING	Severity of the impact based on the impact column value (ref.impact category)
polyphen_pred	STRING	Impact of the SNP as given by PolyPhen (`VEP only`)<br>benign, possibly_damaging, probably_damaging, unknown
polyphen_scores	FLOAT	Polyphen score reflecting severity (higher the impact, *higher* the score) (`VEP only`)
sift_pred	STRING	Impact of the SNP as given by SIFT (`VEP only`)<br>neutral, deleterious
sift_scores	FLOAT	SIFT prob. scores reflecting severity (Higher the impact, *lower* the score) (`VEP only`)

2.8.3 The `samples` table

column name	type	notes
sample_id	INTEGER	PRIMARY_KEY
name	STRING	Sample names
family_id	INTEGER	Family ids for the samples [User defined, default: NULL]
paternal_id	INTEGER	Paternal id for the samples [User defined, default: NULL]
maternal_id	INTEGER	Maternal id for the samples [User defined, default: NULL]
sex	STRING	Sex of the sample [User defined, default: NULL]
phenotype	STRING	The associated sample phenotype [User defined, default: NULL]
ethnicity	STRING	The ethnic group to which the sample belongs [User defined, default: NULL]

2.8.4 Details of the `impact` and `impact_severity` columns

impact severity	impacts
HIGH	• exon_deleted<br>• frame_shift<br>• splice_acceptor<br>• splice_donor<br>• start_loss<br>• stop_gain<br>• stop_loss<br>• non_synonymous_start
MED	• non_syn_coding<br>• inframe_codon_gain<br>• inframe_codon_loss<br>• inframe_codon_change<br>• codon_change_del<br>• codon_change_ins<br>• UTR_5_del<br>• UTR_3_del<br>• other_splice_variant<br>• mature_miRNA<br>• regulatory_region<br>• TF_binding_site<br>• regulatory_region_ablation<br>• regulatory_region_amplification<br>• TFBS_ablation<br>• TFBS_amplification
LOW	• synonymous_stop<br>• synonymous_coding<br>• UTR_5_prime<br>• UTR_3_prime<br>• intron<br>• CDS<br>• upstream<br>• downstream<br>• intergenic<br>• intragenic<br>• gene<br>• transcript<br>• exon<br>• start_gain<br>• synonymous_start<br>• intron_conserved<br>• nc_transcript<br>• NMD_transcript<br>• transcript_codon_change<br>• incomplete_terminal_codon<br>• nc_exon<br>• transcript_ablation<br>• transcript_amplification<br>• feature elongation<br>• feature truncation

2.8.5 The resources table

Establishes provenance of annotation resources used to create a Gemini database.

column name	type	notes
name	STRING	Name of the annotation type
resource	STRING	Filename of the resource, with version information

2.8.6 The version table

Establishes which version of `gemini` was used to create a database.

column name	type	notes
version	STRING	What version of gemini was used to create the DB.

2.9 Using the GEMINI API

2.9.1 The GeminiQuery class

class `gemini.`**`GeminiQuery`**(*db*)

An interface to submit queries to an existing Gemini database and iterate over the results of the query.

We create a GeminiQuery object by specifying database to which to connect:

```
from gemini import GeminiQuery
gq = GeminiQuery("my.db")
```

We can then issue a query against the database and iterate through the results by using the `run()` method:

```
gq.run("select chrom, start, end from variants")
for row in gq:
    print row
```

Instead of printing the entire row, one access print specific columns:

```
gq.run("select chrom, start, end from variants")
for row in gq:
    print row['chrom']
```

Also, all of the underlying numpy genotype arrays are always available:

```
gq.run("select chrom, start, end from variants")
for row in gq:
    gts = row.gts
    print row['chrom'], gts
    # yields "chr1" ['A/G' 'G/G' ... 'A/G']
```

The `run()` methods also accepts genotype filter:

```
query = "select chrom, start, end" from variants"
gt_filter = "gt_types.NA20814 == HET"
gq.run(query)
for row in gq:
    print row
```

Lastly, one can use the `sample_to_idx` and `idx_to_sample` dictionaries to gain access to sample-level genotype information either by sample name or by sample index:

```
# grab dict mapping sample to genotype array indices
smp2idx = gq.sample_to_idx

query  = "select chrom, start, end from variants"
gt_filter  = "gt_types.NA20814 == HET"
gq.run(query, gt_filter)

# print a header listing the selected columns
print gq.header
for row in gq:
    # access a NUMPY array of the sample genotypes.
    gts = row['gts']
    # use the smp2idx dict to access sample genotypes
    idx = smp2idx['NA20814']
    print row, gts[idx]
```

run (*query*, *gt_filter=None*, *show_variant_samples=False*)
Execute a query against a Gemini database. The user may specify:

1.(reqd.) an SQL *query*.

2.(opt.) a genotype filter.

header
Return a header describing the columns that were selected in the query issued to a GeminiQuery object.

sample2index
Return a dictionary mapping sample names to genotype array offsets:

```
gq = GeminiQuery("my.db")
s2i = gq.sample2index

print s2i['NA20814']
# yields 1088
```

index2sample
Return a dictionary mapping sample names to genotype array offsets:

```
gq = GeminiQuery("my.db")
i2s = gq.index2sample

print i2s[1088]
# yields "NA20814"
```

2.10 Acknowledgements

GEMINI is developed by Uma Paila and Aaron Quinlan in the Quinlan laboratory at the University of Virginia. Substantial contributions to the design, functionality, and code base have been made by the following:

- Brad Chapman, HSPH
- Rory Kirchner, HSPH
- Oliver Hofmann, HSPH

2.11 Release History

2.11.1 0.3.0b

1. Improved speed for adding custom annotations.
2. Added GERP conserved elements.
3. Optionally addition of GERP conservation scores at base pair resolution.
4. Move annotation files to Amazon S3.

2.12 F.A.Q.

2.12.1 Does GEMINI work with non-human genomes?

Currently, no. However, we recognize that the GEMINI framework is suitable to genetic research in other organisms. This may be a focus of future work.

2.12.2 What versions of the human genome does GEMINI support?

Currently, we support solely build 37 of the human genome (a.k.a, hg19). We intend to support forthcoming versions of the human genome in future releases.

2.12.3 How can I use PLINK files with GEMINI?

Many datasets, especially those derived from GWAS studies, are based on SNP genotyping arrays, and are thus stored in the standard PLINK formats. While GEMINI only supports VCF input files, it is relatively straightforward to convert PLINK datasets to VCF with the PLINK/SEQ toolkit.

1. First, load the PLINK BED file into a new PLINK/SEQ project using the instructions found in the "Load a PLINK binary fileset" section here.

2. Next, use PLINK/SEQ to convert the project to VCF using the instructions found here.

At this point, you should have a VCF file that is compatible with GEMINI.

Alternatively, in his bcbio project, Brad Chapman has written a convenient script for directly converting PLINK files to VCF. Below is an example of how to use this script.

```
$ plink_to_vcf.py <ped file> <map file> <UCSC reference file in 2bit format)
```

PYTHON MODULE INDEX

g

gemini, 43

INDEX

G

gemini (module), 43
GeminiQuery (class in gemini), 43

H

header (gemini.GeminiQuery attribute), 44

I

index2sample (gemini.GeminiQuery attribute), 44

R

run() (gemini.GeminiQuery method), 44

S

sample2index (gemini.GeminiQuery attribute), 44
